# MVA-based SARS-CoV-2 vaccine candidates encoding different spike protein conformations induce distinct early transcriptional responses which may impact subsequent adaptive immunity

**DOI:** 10.3389/fimmu.2024.1500615

**Published:** 2024-12-19

**Authors:** Ilka Grewe, Monika Friedrich, Marie-Louise Dieck, Michael Spohn, My Linh Ly, Verena Krähling, Leonie Mayer, Sibylle C. Mellinghoff, Monika Rottstegge, Rebekka Kraemer, Asisa Volz, Stephan Becker, Anahita Fathi, Christine Dahlke, Leonie M. Weskamm, Marylyn M. Addo

**Affiliations:** ^1^ Institute for Infection Research and Vaccine Development (IIRVD), Center for Internal Medicine, University Medical Center Hamburg-Eppendorf, Hamburg, Germany; ^2^ Department for Clinical Immunology of Infectious Diseases, Bernhard Nocht Institute for Tropical Medicine, Hamburg, Germany; ^3^ First Department of Medicine, Division of Infectious Diseases, University Medical Center Hamburg-Eppendorf, Hamburg, Germany; ^4^ German Center for Infection Research, Partner Site Hamburg-Lübeck-Borstel-Riems, Hamburg, Germany; ^5^ Research Institute Children’s Cancer Center Hamburg, Hamburg, Germany; ^6^ Department of Pediatric Hematology and Oncology, University Medical Center Hamburg-Eppendorf, Hamburg, Germany; ^7^ Bioinformatics Core, University Medical Center Hamburg-Eppendorf, Hamburg, Germany; ^8^ Institute of Virology, Philipps University Marburg, Marburg, Germany; ^9^ German Center for Infection Research, Partner Site Gießen-Marburg-Langen, Marburg, Germany; ^10^ Institute of Translational Research, Cluster of Excellence for Aging Research (CECAD), Faculty of Medicine and University Hospital of Cologne, University of Cologne, Cologne, Germany; ^11^ Center for Integrated Oncology Aachen Bonn Cologne Düsseldorf (CIO ABCD), Department I of Internal Medicine, Faculty of Medicine and University Hospital of Cologne, University of Cologne, Cologne, Germany; ^12^ Institute of Clinical Molecular Biology, Christian-Albrechts-University and University Medical Center Schleswig-Holstein, Kiel, Germany; ^13^ Institute of Virology, University of Veterinary Medicine Hannover, Hanover, Germany; ^14^ German Center for Infection Research, Partner Site Hannover-Braunschweig, Hannover, Germany

**Keywords:** SARS-CoV-2, COVID-19, modified vaccinia virus Ankara, systems vaccinology, spike protein, transcriptome, T follicular helper cells, innate immunity

## Abstract

**Introduction:**

Vaccine platforms such as viral vectors and mRNA can accelerate vaccine development in response to newly emerging pathogens, as demonstrated during the COVID-19 pandemic. However, the differential effects of platform and antigen insert on vaccine immunogenicity remain incompletely understood. Innate immune responses induced by viral vector vaccines are suggested to have an adjuvant effect for subsequent adaptive immunity. Integrating data on both innate and adaptive immunity, systems vaccinology approaches can improve the understanding of vaccine-induced immune mechanisms.

**Methods:**

Two vaccine candidates against SARS-CoV-2, both based on the viral vector Modified Vaccinia virus Ankara (MVA) and encoding the native (MVA-SARS-2-S) or prefusion-stabilized spike protein (MVA-SARS-2-ST), were evaluated in phase 1 clinical trials (ClinicalTrials.gov: NCT04569383, NCT04895449). Longitudinal dynamics of innate and early adaptive immune responses induced by vaccination in SARS-CoV-2-naïve individuals were analyzed based on transcriptome and flow cytometry data, in comparison to the licensed ChAd and mRNA vaccines.

**Results:**

Compared to MVA-SARS-2-S, MVA-SARS-2-ST (encoding the prefusion-stabilized spike protein) induced a stronger transcriptional activation early after vaccination, as well as higher virus neutralizing antibodies. Positive correlations were observed between innate and adaptive immune responses induced by a second MVA-SARS-2-ST vaccination. MVA-, ChAd- and mRNA-based vaccines induced distinct immune signatures, with the overall strongest transcriptional activation as well as monocyte and circulating T follicular helper (cTFH) cell responses induced by ChAd.

**Discussion:**

Our findings suggest a potential impact of the spike protein conformation not only on adaptive but also on innate immune responses. As indicated by positive correlations between several immune parameters induced by MVA-SARS-2-ST, the distinct transcriptional activation early after vaccination may be linked to the induction of classical monocytes and activation of cTFH1 cells, which may in turn result in the superior adaptive immunogenicity of MVA-SARS-2-ST, compared to MVA-SARS-2-S. Overall, our data demonstrate that both the vaccine platform and antigen insert can affect innate immune responses and subsequent vaccine immunogenicity in humans.

## 1 Introduction

Rapid vaccine development is imperative to combat epidemic or pandemic outbreaks, as recently observed during the COVID-19 pandemic. One approach to accelerate the response to newly emerging pathogens are vaccine platforms which can be easily adapted to encode new antigens (1, 2). Modified Vaccinia virus Ankara (MVA), an attenuated non-replicating poxviral vector, was originally developed as a third-generation smallpox vaccine and was recently also licensed against mpox (3, 4). In its recombinant form, it represents a promising and safe vaccine platform (5). A recombinant MVA vaccine against Ebola virus (MVA-BN-Filo) was the second viral vector vaccine to ever be licensed and is currently recommended to be applied in a heterologous vaccine regimen (6, 7). Encouraging results have also been achieved in recent clinical trials investigating MVA-MERS-S, an MVA-based vaccine against the Middle East respiratory syndrome coronavirus (8, 9), demonstrating favorable safety profiles and long-lasting immunogenicity.

In response to the COVID-19 pandemic, two recombinant MVA vaccine candidates, encoding different conformations of the SARS-CoV-2 spike protein, were evaluated in phase 1 clinical trials (10, 11). The spike protein consists of the S1 and the S2 subunit, which engage with the host cell receptor angiotensin-converting enzyme 2 and mediate the fusion of the viral and host cell membranes (12, 13). It represents an important vaccine target antigen since neutralizing antibodies against the spike protein can block virus entry into the host cell (14). We first investigated the safety and immunogenicity of a recombinant MVA viral vector vaccine expressing the native, full-length spike protein (MVA-SARS-2-S, hereafter MVA-S) in a phase 1 clinical trial (ClinicalTrials.gov: NCT04569383), after the vaccine had been shown to be immunogenic in mice and hamsters (15). Since further preclinical investigations suggested increased immunogenicity of a recombinant MVA vaccine expressing a prefusion-stabilized version of the SARS-CoV-2 spike protein with an inactivated S1/S2 cleavage site and K986P and V987P mutations (MVA-SARS-2-ST, hereafter MVA-ST), we then investigated this optimized vaccine candidate in a phase 1 clinical trial (ClinicalTrials.gov: NCT04895449) (10).

The clinical evaluation revealed the optimized MVA-ST vaccine to be more immunogenic than MVA-S, as reflected in the spike-specific IgG, B cell, and T cell responses (11). In particular, MVA-ST induced higher S1-specific responses compared to MVA-S, while the latter induced responses skewed towards the S2 subunit, underlining the importance of the conformation of the SARS-CoV-2 spike protein used as vaccine antigen (11). The licensed viral vector vaccine ChAdOx1 nCov-19 (in this manuscript referred to as ChAd), which is based on a replication-deficient Chimpanzee Adenovirus vector and also encoding for the native spike protein, and the mRNA vaccines BNT162b2 and mRNA-1273, encoding for the prefusion-stabilized spike protein, were shown to induce higher humoral and cellular immune responses in a direct comparison to both of the recombinant MVA-based SARS-CoV-2 vaccines (11).

The underlying mechanisms of the differential immune responses towards distinct vaccine platforms, as well as to antigen inserts encoding different conformations of the spike protein, are not fully elucidated to date. Especially innate and T follicular helper (TFH) cell responses are often not included in the immune monitoring of clinical vaccine trials, as they require frequent sampling early after vaccination. However, these parameters of the early immune response are crucial for vaccine efficacy, as they mediate the induction of a potent adaptive immune response. TFH cells are a CXCR5+ subset of CD4+ cells, that localizes in germinal centers of lymphoid tissue and regulates clonal selection and differentiation of memory B cells and plasmablasts and is therefore crucial for the development of protective immunity following vaccination (16, 17). Circulating T follicular helper (cTFH) cells, a correlate of germinal center TFH cells that can be detected in the blood, have frequently been found to correlate with vaccine-induced antibody responses (16–19). cTFH cells can be classified by their expression of CXCR3 and CCR6 into cTFH1 (CXCR3+ CCR6-), cTFH2 (CXCR3- CCR6-) and cTFH17 (CXCR3- CCR6+) cells. The role of each subset in B cell support has been discussed controversially, but several studies are pointing towards a role of cTFH1 cells correlating with B cell and antibody responses following vaccination (18, 20–22). A study comparing a protein vaccine and a heterologous vector vaccine regimen based on ChAd and MVA against malaria found that the protein vaccine induced a generally stronger cTFH2 skewed response, while the ChAd/MVA regimen induced a stronger cTFH1 response (23).

A comprehensive side-by-side comparison of innate and adaptive immune responses induced by different SARS-CoV-2 vaccine platforms in humans can give valuable insights into the generation of protective immunity and therefore inform future vaccine design (24). In the context of viral vector vaccines, the innate immune response towards the vector itself is of additional interest as it can increase immunogenicity, potentially reducing the need for a vaccine adjuvant (25).

To date, no studies have systematically compared gene expression, innate and cTFH responses following recombinant MVA-based vaccination to ChAd- and mRNA-based vaccines. In addition, in-human studies comparing immune responses induced by different vaccines based on the same viral vector backbone but encoding different conformations of the same antigen are scarce. In the present study, we present data from a systems vaccinology study including a longitudinal analysis of innate and early adaptive immune responses induced by the MVA-S (native spike) and MVA-ST (prefusion-stabilized spike) vaccine candidates evaluated in human phase 1 clinical trials, in direct comparison to the licensed ChAd and mRNA vaccines in SARS-CoV-2-naïve individuals. Our study gives insight into the impact of both vaccine platform and vaccine antigen on early immune responses after vaccination.

## 2 Methods

### 2.1 Vaccines

MVA-S (MVA-SARS-2-S) and MVA-ST (MVA-SARS-2-ST) are two vaccine candidates which are based on recombinant MVA vectors. MVA-S encodes the native full-length spike protein of SARS-CoV-2, while MVA-ST encodes a prefusion-stabilized spike protein with an inactivated S1/S2 furin cleavage site (10, 11, 15). The licensed ChAd vaccine ChAdOx1 nCoV-19 (Vaxzevria) is based on the modified chimpanzee adenovirus ChAdOx1 vector, encoding the full-length spike protein and a tissue plasminogen activator leader sequence (26). The licensed mRNA vaccines BNT162b2 (Comirnaty) and mRNA-1273 (Spikevax) are lipid-nanoparticle-formulated nucleoside-modified mRNA vaccines encoding the prefusion-stabilized spike protein (27). All four vaccines are monovalent, based on the spike protein of wild type SARS-CoV-2.

### 2.2 Study approval

The phase 1 clinical trials investigating the vaccine candidates MVA-S and MVA-ST were reviewed and approved by the National Competent Authority (Paul-Ehrlich-Institute, EudraCT numbers 2020-003875-16 and 2021-000548-23) and the Ethics Committee of the Hamburg Medical Association (reference numbers 2020-10164-AMG-ff; 2021-100621-AMG-ff), conducted under the sponsorship of the University Medical Center Hamburg-Eppendorf (Hamburg, Germany) in accordance with ICH-GCP and the EU directives 2001/20/EC and 2001/83/EC, and are registered at ClinicalTrials.gov. (NCT04569383; NCT04895449). The monitoring of immune responses following vaccination with the licensed ChAd and mRNA vaccines was approved by the Ethics Committee of the Hamburg Medical Association (reference number: 2020-10376-BO-ff). Written informed consent was obtained from all participants.

### 2.3 Study cohorts

NCT04569383 was a phase 1 clinical trial conducted between October 2020 and August 2021 to evaluate the MVA-S vaccine candidate in 30 seronegative individuals divided into two ascending dose groups. Participants were enrolled at the University Medical Center Hamburg-Eppendorf and received two single injections 28 days apart of either a low dose of 1 × 10^7^ ± 0.5 log IU (N = 15) or a high dose of 1 × 10^8^ ± 0.5 log IU (N = 15). In this study, we included a subgroup of participants (N = 12), of whom six participants received the low dose and six received the high dose vaccine.

NCT04895449 was a phase 1b clinical trial conducted between July 2021 and November 2022 to evaluate the MVA-ST vaccine candidate in seronegative individuals (Part A) and in individuals who had previously received two doses of the BNT162b2 vaccine (Part B). In Part A, participants received two single injections 28 days apart, either a low dose of 1 × 10^7^ ± 0.5 log IU (N = 8) or a middle dose of 5 × 10^7^ ± 0.5 log IU (N = 7) and were enrolled at the University Medical Center Hamburg-Eppendorf and the University of Cologne. All participants of Part A (N = 15) were included into this study, while Part B of the clinical trial was not included in the present manuscript.

The mRNA and ChAd/mRNA control cohorts received licensed vaccines known to be immunogenic and were used as a benchmark for the evaluation of the MVA-based vaccines. The participants received either two doses of mRNA vaccine 21 days apart (N = 10), or one dose ChAd plus one dose mRNA vaccine 84 days apart (N = 8), respectively. The participants were enrolled at the University Medical Center Hamburg-Eppendorf between December 2020 and March 2021.

Individuals with self-reported prior SARS-CoV-2 exposure, clinical evidence of COVID-19-like symptoms or positive SARS-CoV-2 antigen and/or PCR test were excluded. If participants acquired a SARS-CoV-2 infection during the study period, subsequent immunogenicity time points were excluded from the analyses in this manuscript.

**Figure 1:**
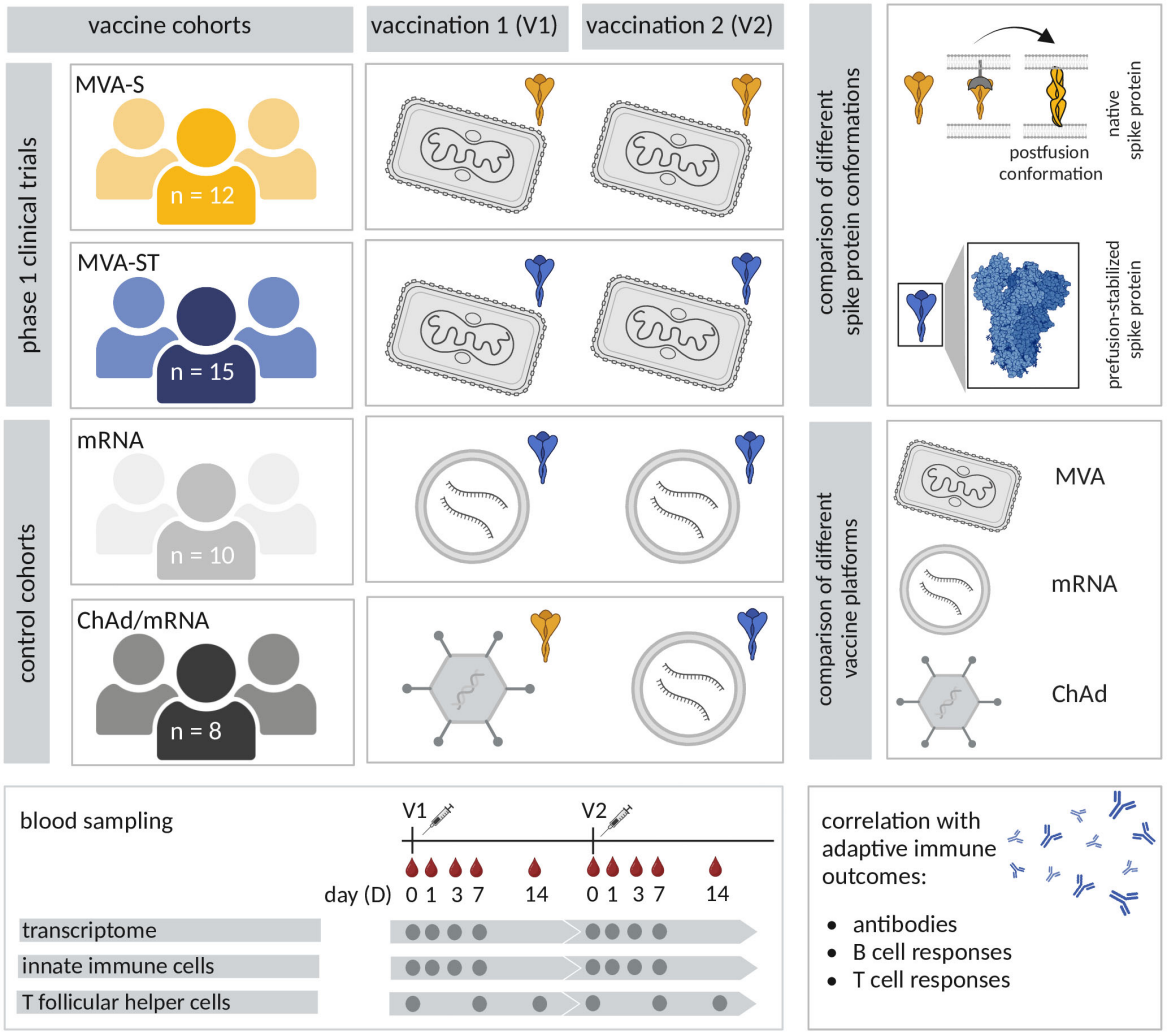
Study overview. In four study cohorts, participants received two vaccinations with different SARS-CoV-2 vaccines. Participants of the first two cohorts received the recombinant MVA-based vaccine candidates MVA-S (MVA-SARS-2-S, yellow) and MVA-ST (MVA-SARS-2-ST, blue) in the scope of two phase 1 clinical trials. The control cohorts received different combinations of licensed vaccines, used as benchmarks for vaccine immunogenicity. One control cohort received two vaccinations with the licensed mRNA vaccine BNT162b2 (light grey). Participants of the fourth cohort (dark grey) received a dose of the licensed ChAd vaccine (ChAdOx1 nCov-19), followed by a dose of a licensed mRNA vaccine (BNT162b2 or mRNA-1273). The different vaccines encode either the native spike protein (yellow) or the prefusion-stabilized spike protein (blue). Blood samples were collected prior to vaccination (D0) and on D1, D3, D7 and D14 after the first and the second vaccination (V1 and V2). Transcriptional responses as well as innate and spike-specific circulating T follicular helper (cTFH) cell responses were analyzed and evaluated for correlations with adaptive immune outcomes (spike-specific neutralizing and binding antibodies, B and T cell responses).

An overview of all study cohorts is given in **Figure 1**. Baseline characteristics, the interval between vaccinations, and the number of samples included in the different analyses according to cohort and time point, are reported in **Supplementary Tables 1**-**6**.

### 2.4 Blood sampling and processing

A total of 384 peripheral blood samples were obtained from 45 donors. Whole blood was collected in EDTA vacutainers at day 0 (D0, baseline before vaccination), day 1 (D1), day 3 (D3), day 7 (D7) and day 14 (D14) after the first and second vaccination (V1, V2). After centrifugation, plasma was removed and stored at −80°C. PBMCs were isolated by density-gradient centrifugation using Ficoll-Histopaque (Sigma) or SepMate™ (Stemcell), cryopreserved and stored in a gas phase liquid nitrogen tank. Serum was collected at V1D0 and V2D14 using S-Monovettes® containing gel with clotting activators and stored at −20°C. Additionally, whole blood was collected directly into PAXgene RNA tubes (PreAnalytiX, QIAGEN) for bulk RNA sequencing on D0, D1, D3 and D7 post V1 and V2. After keeping the samples for 3-4h at room temperature (RT), samples were stored at -80°C until RNA isolation.

### 2.5 Gene expression analysis

Gene expression was analyzed on D0, D1, D3 and D7 after each vaccination. RNA isolation from thawed PAXgene RNA tubes was performed following the manufacturer’s instructions (PAXgene Blood RNA Kit Handbook, version 2, 2015) using the PAXgene blood RNA kit (PreAnalytiX, QIAGEN), including optional DNase digestion. Extracted RNA from all samples was stored at -80°C.

RNA-seq libraries were generated using the TruSeq stranded mRNA Kit according to the manufacturer’s guidelines. Library size and quality were assessed using a BioAnalyzer high-sensitivity chip (Agilent Technologies), and concentrations were measured with the Qubit 2.0 fluorometer (Life Technologies). Multiplex sequencing was performed on the NovaSeq S4 (2x100bp run). Samples with RNA integrity number (RIN) < 8, insufficient library concentration, or sequencing depth below 25 million reads were excluded from the analysis.

Quality control of raw reads was conducted using FastQC v0.11.8 and MultiQC v1.9. Removal of low-quality read ends and adapters was carried out using fastp v0.23.2 prior to read mapping and gene-wise counting with STAR v.2.7.10a. Ensembl genome GRCh38 and Annotation version 110 were utilized for this purpose. Normalization of raw counts and identification of differentially expressed genes (DEGs) were performed using DESeq2 v1.36.0, with all time points compared to V1D0. A gene was considered differentially expressed with an absolute log2 fold change > 1 and FDR < 0.1.

### 2.6 Flow cytometry analysis

Innate immune cells were assessed on D0, D1, D3 and D7 after each vaccination. Cryopreserved PBMCs were thawed, distributed into a 96-well V-bottom plate at a number of 1x10^6^ cells per well and stained. For the staining of subsets and activation status of monocytes and dendritic cells (DCs), PBMCs were incubated for 15min at 37°C in 100 µl staining buffer containing PBS, 1% FBS, 1mM EDTA, anti-CD3-APC/Cy7 (1 µl), anti-CD19-APC/Cy7 (1 µl), anti-CD16-BV510 (3 µl), anti-CD14-BV711 (3 µl), anti-CD303-PerCP/Cy5.5 (3 µl), anti-CD11c-APC (1 µl), anti-HLADR-BV785 (3 µl), anti-CD197 (CCR7)-BV605 (3µL), anti-CD40-PE (1 µl), anti-CD1c-FITC (2 µl), anti-CD141-PE/Cy7 (3 µl), anti-CD11b-BUV395 (0.5 µl) and Zombie NIR™ fixable viability stain (0.2 µl).

cTFH cell subsets and activation status were analyzed on D0, D7 and D14 following each vaccination. Following thawing of cryopreserved PBMCs, the cells were rested for 90 min at 37°C and 5% CO_2_ in R10 (RPMI containing 1% Penicillin/Streptomycin and 10% FBS) and subsequently distributed in a 96 well V-bottom plate (2x10^6^ cells/well) in duplicates. In order to analyze cTFH cells in their ex vivo state and after restimulation with SARS-CoV-2 spike overlapping peptide pools, one replicate of each sample was incubated with R10, and the other replicate was stimulated with overlapping peptide pools M1-M4, spanning the S1 and S2 subunit of the SARS-CoV-2 spike protein (final concentration: 1 µg/ml; for peptide sequences see **Supplementary Table 7**) in a final volume of 150 µl per well for 16 h at 37°C and 5% CO_2._ Both replicates – stimulated and unstimulated – were incubated in the presence of 1 µl anti-CCR6-PE/Dazzle per well. Subsequently, cells were stained by incubating them for 15 min at 37°C in 100 µl of staining buffer (PBS, 1% FBS, 1mM EDTA) containing anti-CD4-PerCP/Cy5.5 (1 µl), anti-CD45RO-FITC (1 µl), anti-ICOS-PE/Cy7 (0.2 µl), anti-CD25-PE (1 µl), anti-CD19-APC/Cy7 (1 µl), anti-CD14-APC/Cy7 (1 µl), anti-CD3-AF700 (1 µl), anti-PD1-APC (1 µl), anti-CD127-BV785 (1 µl), anti-CXCR5-BV711 (1 µl), anti-CXCR3-BV650 (1 µl), anti-CD38-BUV605 (1 µl), anti-CCR7-BV421 (1 µl), anti-HLADR-BUV737 (1 µl), anti-CD8-BUV395 (1 µl), and Zombie NIR™ fixable viability stain (0.2 µl).

Following the staining of surface markers for either innate or cTFH cells, the PBMCs were fixed in 4% PFA for 15 min at RT, washed, resuspended in 100 µl of staining buffer and acquired using an LSR Fortessa™ (BD). Data analysis was performed using the FlowJo software (v.10.7.2, Becton Dickinson, Franklin Lakes). Gating strategies are shown in **Supplementary Figures 1**, **2**. Fluorescence minus one controls were used to set the gates for activation markers.

### 2.7 SARS-CoV-2 VNT_100_

A virus neutralization test (VNT_100_) was used to assess the serum neutralization capacity against wild type SARS-CoV-2 as described and reported previously (28). Following heat-inactivation for 30 min at 56°C and sample dilution in a two-fold dilution series (1:4–1:512), 100 plaque-forming units (PFU) of SARS-CoV-2 (German isolate BavPat1/2020; European Virus Archive Global #026 V-03883 (Genbank: MZ558051.1)) were added and samples were incubated for 1 h at 37°C. Subsequently, 2 × 10^4^ Vero C1008 cells (ATCC, Cat. No. CRL-1586, RRID: CVCL_0574) were added. At day 4 post infection, cytopathic effects were evaluated. Neutralization was defined as the absence of cytopathic effects. The reciprocal neutralization titer was calculated from the highest serum dilution without cytopathic effects (geometric mean based on three replicates). The lower limit of detection (LLOD) was defined as a reciprocal titer of 8, corresponding to the first dilution of the respective serum.

### 2.8 Statistical analysis and visualization

DEGs were identified by calculating p-values using Wald test, with adjustments for multiple comparisons using the Benjamini & Hochberg correction. Subsequently, gene expression data were analyzed using Ingenuity Pathway Analysis (v.111725566, QIAGEN). Data analysis and visualization were conducted with GraphPad Prism (v.9.5.1, Dotmatics, Boston, USA) and Rstudio (2023.06.1, R v.4.2.0, The R Foundation, Vienna, Austria). Apart from gene expression analysis, statistical significance tests were not conducted due to the limited statistical power resulting from the small sample size and the high number of parameters assessed. Correlation analyses were performed in R using Spearman’s non-parametric correlation. Descriptive statistics of all reported parameters are reported in **Supplementary Tables 8**-**30**.

## 3 Results

### 3.1 Study overview

To gain insight into the early immune responses induced by the MVA-S and MVA-ST vaccines, as well as by the licensed mRNA and ChAd vaccines, we collected blood from the study participants of all cohorts at time points D0 (baseline), D1, D3, D7 and D14 following two vaccinations (V1, V2) against SARS-CoV-2. Whole blood transcriptome, as well as innate and cTFH cell activation were analyzed longitudinally. To identify early immune parameters potentially related to the differential immunogenicity of the MVA-S and MVA-ST vaccines, based on the same viral vector but encoding different conformations of the SARS-CoV-2 spike protein, we performed a correlation analysis between early immune parameters and adaptive immune outcomes after completion of the vaccination series (V2D14). The study design and cohorts are depicted in **Figure 1**, including an overview of the different vaccination schedules, vaccine platforms and antigens, sampling time points and analyzed parameters. Baseline characteristics of study participants are listed in **Supplementary Table 1**.

### 3.2 MVA-S and MVA-ST vaccination induce differential transcriptional responses

To profile vaccine-induced changes in the gene expression, we performed a whole blood transcriptome analysis based on PaxGene tubes collected at baseline (D0), as well as on D1, D3 and D7 following each vaccination (V1, V2). Overall, we integrated transcriptional data of 317 samples from 43 adults, vaccinated with one of four different SARS-CoV-2 vaccination regimens (MVA-S, MVA-ST, mRNA and ChAd/mRNA).

**Figure 2:**
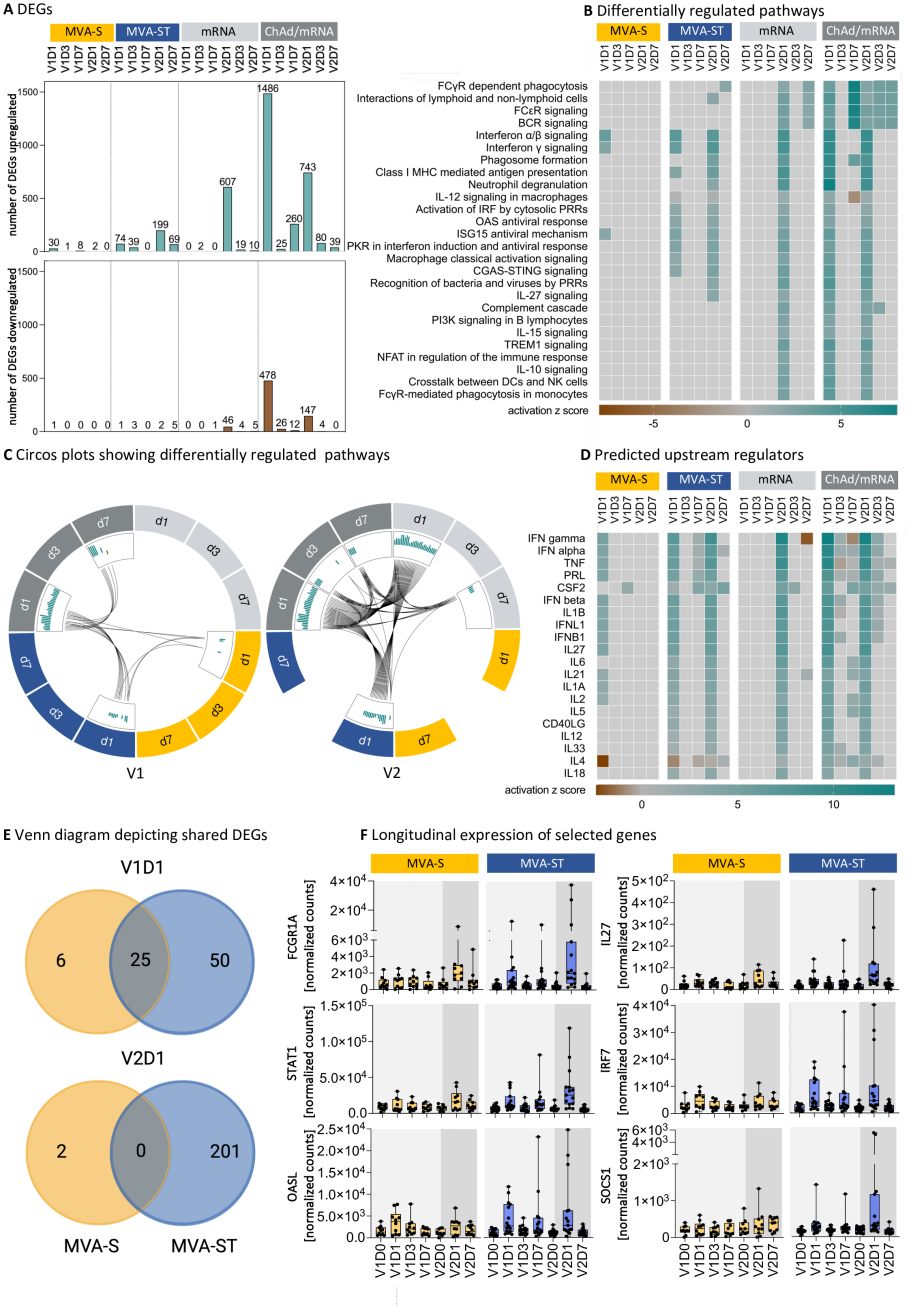
MVA-S and MVA-ST vaccination induce differential transcriptional responses. **(A)** Number of upregulated or downregulated differentially expressed genes (DEGs) on D1, D3 and D7 after each vaccination with MVA-S, MVA-ST, mRNA or ChAd/mRNA, defined as genes with a false discovery rate (FDR) <0.1 and an absolute log fold change ≥1 compared to V1D0. **(B)** Canonical pathways involved in the immune system, which are up- or downregulated with -log (FDR) >5 and absolute z-score >2 for at least one time point in one cohort, as analyzed by Ingenuity Pathway Analysis (IPA, Qiagen). The number of DEGs in each pathway is shown in **Supplementary Tables 31**-**34**. **(C)** Circos plots connecting canonical pathways, which are significantly up- (green) or downregulated (brown) at more than one time point or in more than one group. **(D)** Ingenuity upstream regulator cytokine analysis using DEGs with FDR <0.1 and an absolute log fold change ≥1 compared to V1D0. The heatmap shows the top upstream cytokines with a -log (FDR) >5 and absolute z-score >2 for at least one time point in one cohort. **(E)** Venn diagrams showing shared DEGs on V1D1 and V2D1 following MVA-S and MVA-ST vaccination. All DEGs following MVA-S and MVA-ST vaccination are listed in **Supplementary Tables 35**, **36**, respectively. **(F)** Longitudinal expression of selected genes involved in canonical signaling pathways, which are differentially up- or downregulated on at least one time point following MVA-S or MVA-ST vaccination. Depicted are the normalized counts calculated by variance stabilizing transformation (VST). Boxplots depict median and interquartile range, dots resemble individual data points. Longitudinal expression levels of all DEGs of the MVA-S/ST cohorts, according to the canonical signaling pathways shown in **(C)**, are shown in **Supplementary Figures 3**-**18**.

DEGs were identified after vaccination with both MVA-based vaccine candidates. However, we observed a stronger transcriptional response after the first vaccination with MVA-ST compared to MVA-S, with 75 compared to 31 DEGs on V1D1 and 42 compared to 8 DEGs on V1D7, respectively (**Figure 2A**). Also, the transcriptional response after the second vaccination with MVA-ST was stronger compared to MVA-S, with 201 compared to 2 DEGs on V2D1 and 74 compared to 0 DEGs on V2D7, respectively. In order to better understand the immune responses induced by different vaccine platforms, we compared transcriptional responses of both MVA-based vaccine candidates with the licensed ChAd and mRNA vaccines. We observed a strong transcriptional response after ChAd vaccination with 1964, 51 and 272 DEGs at V1D1, V1D3 and V1D7, respectively. In the mRNA cohort, we detected only 2 DEGs on V1D3 and 1 DEG on V1D7 following the first vaccination. These findings are similar to a study by Ryan et al., which did not detect any DEGs on D6 following mRNA vaccination (29). Following the second vaccination, numerous upregulated DEGs were detected in both the mRNA cohort (V2D1 = 653, V2D3 = 23, V2D7 = 15) and the ChAd/mRNA cohort (V2D1 = 890, V2D3 = 84, V2D7 = 39) (**Figure 2A**).

In order to identify differentially upregulated pathways, we next performed an ingenuity pathway analysis (**Figure 2B**). At V1D1 we observed an upregulation of the Interferon alpha/beta signaling and Interferon gamma signaling for both MVA-S and MVA-ST. For MVA-ST, an additional upregulation of multiple pathways of the innate immune response was observed, including the classical activation of macrophages, activation of IRF by cytosolic pattern-recognition receptors (PRRs), the cGAS-STING pathway and the human oligoadenylate synthetase (OAS) antiviral response, as well as MHC class I mediated antigen presentation. The same pathways were also upregulated at V1D1 after ChAd vaccination. In addition, ChAd vaccination induced an upregulation of the complement cascade and several pathways mediating adaptive immunity (interactions of lymphoid and non-lymphoid cells, B cell receptor (BCR) signaling and PI3K signaling in B lymphocytes). In contrast, a single mRNA vaccination did not lead to an upregulation of any of these immune pathways. While there were no pathways upregulated on V1D7 after vaccination with MVA-S or MVA-ST, an upregulation of BCR signaling was observed on V1D7 after ChAd vaccination. Following the second vaccination, we did not detect any differentially regulated pathways in the MVA-S cohort, while there was a number of upregulated pathways in the MVA-ST cohort. Besides the pathways identified at V1D1, the pathways upregulated at V2D1 in the MVA-ST cohort included the interactions between lymphoid and non-lymphoid cells, phagosome formation, neutrophil degranulation, the role of PRRs in recognition of bacteria and viruses and IL27 signaling. In addition to the upregulated pathways detected after the second MVA-ST vaccination, the pathways upregulated after the second vaccination in the ChAd/mRNA and mRNA cohorts (V2D1) included the complement cascade, PI3K signaling in B lymphocytes, IL10 and IL15 signaling, and FcγR-mediated phagocytosis. The number of DEGs involved in each of the pathways shown in **Figure 2B** are reported in **Supplementary Tables 31**-**34** for each vaccine cohort. The shared upregulated pathways between the different cohorts and time points are additionally highlighted in the Circos plots (**Figure 2C**). The connecting lines underline that after the first vaccination pathways were mostly shared between MVA-ST and ChAd vaccination, whereas the second vaccination activated an overall higher number of pathways, which were shared between the MVA-ST, ChAd/mRNA and mRNA cohort.

To identify upstream cytokines that could potentially regulate the differential immune responses, we performed an upstream regulator analysis and filtered for predicted upstream cytokines using DEGs with FDR <0.1 and an absolute log fold change ≥1 compared to V1D0 (**Figure 2D**). We identified several members of the Interferon family as predicted upregulated upstream regulators of the observed DEG signature after the first vaccination with both MVA-S and MVA-ST, but only after the second vaccination with MVA-ST. In addition, the upstream regulators predicted for both the first and the second MVA-ST vaccination included cytokines produced by monocytes and macrophages (IL1α, IL1β, IL6, IL18), and molecules involved in the interaction of TFH cells with B cells (IL21, CD40L). For MVA-S, these upstream regulators were only predicted for the response to the first vaccination (IL1α, IL1β, IL21) or not predicted at all (IL6, IL18, CD40L) (**Figure 2E**).

Since MVA-S and MVA-ST are based on the same vector but encode different conformations of the spike protein, one explanation for the observed differences in gene expression could be the different spike protein conformations. Taking a closer look at the DEGs induced by MVA-S and MVA-ST vaccination, we identified 25 DEGs that are shared between the two cohorts at V1D1, and no shared DEGs at V2D1 (**Figure 2E**). The majority of the detected DEGs (V1D1 = 50 DEGs, V2D1 = 201 DEGs) was unique for MVA-ST vaccination. The normalized counts of six selected DEGs, that are involved in the identified upregulated pathways and showed the strongest differences between the MVA-S and MVA-ST cohort at the peak time point, are shown in **Figure 2F**. We observed a stronger upregulation of FCGR1A (Interactions of lymphoid and non-lymphoid cells pathway), STAT1 (CGAS STING pathway and protein kinase R (PKR) in Interferon Induction and Antiviral Response pathway), OASL (OAS antiviral response pathway), IL27 (IL27 signaling pathway), IRF7 (Interferon gamma signaling) and SOCS1 (Macrophage classical activation signaling pathway), among other genes, on V1D1 and V2D1 for MVA-ST compared to MVA-S vaccination.

### 3.3 ChAd- and mRNA-based vaccines induce a strong response of classical monocytes

**Figure 3:**
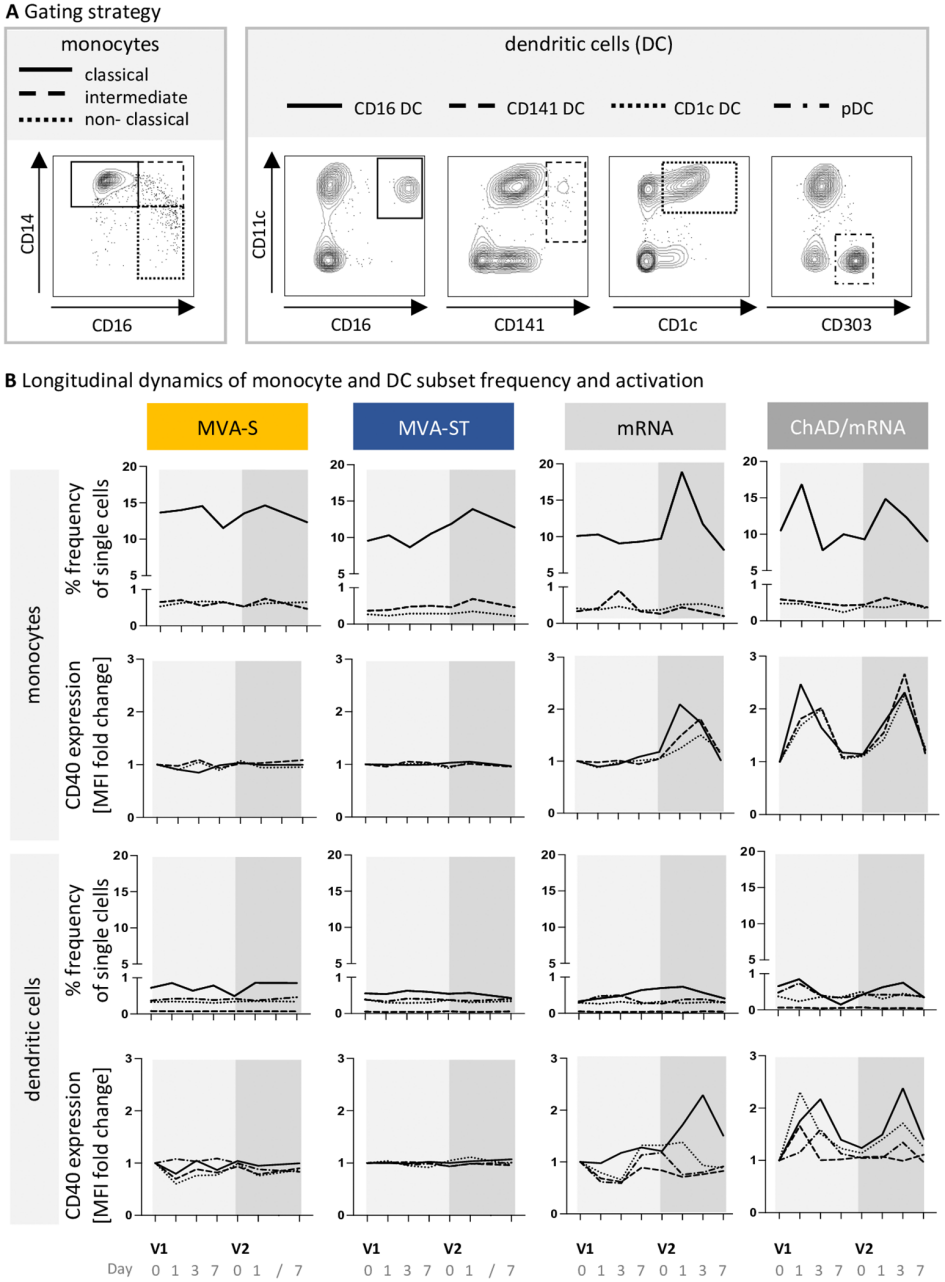
ChAd- and mRNA-based vaccines induce a strong response of classical monocytes. **(A)** Gating schemes used for differentiation of monocyte subsets within HLA-DR+CD11b+ cells, and dendritic cell (DC) subsets within HLA-DR+CD11b- cells, respectively. The complete gating strategy for identification of monocytes and DCs within whole PBMCs, is shown in **Supplementary Figure 1**. **(B)** Longitudinal dynamics of the frequency and activation (CD40 expression) of monocyte and DC subsets at baseline (D0) and on D1, D3 and D7 following each vaccination. Displayed is the median frequency of each subpopulation, and the median fold change of the CD40 expression compared to V1D0. MFI, median fluorescence intensity. Descriptive statistics for all parameters are reported in **Supplementary Tables 8**-**21**.

Building on the observed transcriptional upregulation of innate immune pathways such as the activation of classical macrophages after MVA-ST but not after MVA-S vaccination, we investigated the monocyte-macrophage system in more detail using PBMCs derived from vaccinees at D0, D1, D3, and D7 after each vaccination. Using flow cytometry, different monocyte and DC subsets were analyzed (**Figure 3A**). While a strong response of monocytes was observed after ChAd/mRNA vaccination, including an increase in the frequency of classical monocytes at D1 (median fold change compared to V1D0 (mfc) V1D1 = 1.7, V2D1 = 1.3) and an activation of all investigated monocyte subsets at D1 and D3 as measured by the expression of CD40 [mfc up to 2.5 (V1) and 2.7 (V2)], we only detected a slight increase in the frequency of classical monocytes after the second vaccination with the MVA-based vaccine candidates (mfc MVA-S, V2D1 = 1.1; MVA-ST, V2D1 = 1.2) (**Figure 3B**). Furthermore, an activation of DCs, in particular CD16+ DCs, was observed at D3 after vaccination with ChAd/mRNA (mfc V1D3 = 2.2, mfc V2D3 = 2.4), whereas no activation was observed after vaccination with MVA-S or MVA-ST (mfc < 1.1). Consistent with the absence of transcriptional responses after the first mRNA vaccination, we also did not observe any innate responses after the first vaccination on the PBMC level. However, after the second vaccination with mRNA, an increase in the frequency of classical monocytes (mfc V2D1 = 1.9), as well as an activation of all monocyte subsets [mfc up to 1.1 (V1) and 2.1 (V2)] and CD16+ DCs (mfc V1D3 = 1.2, V2D3 = 2.3) was detected (**Figure 3B**).

### 3.4 ChAd-based primary vaccination induces a stronger cTFH cell response compared to mRNA- and MVA-based vaccination

**Figure 4:**
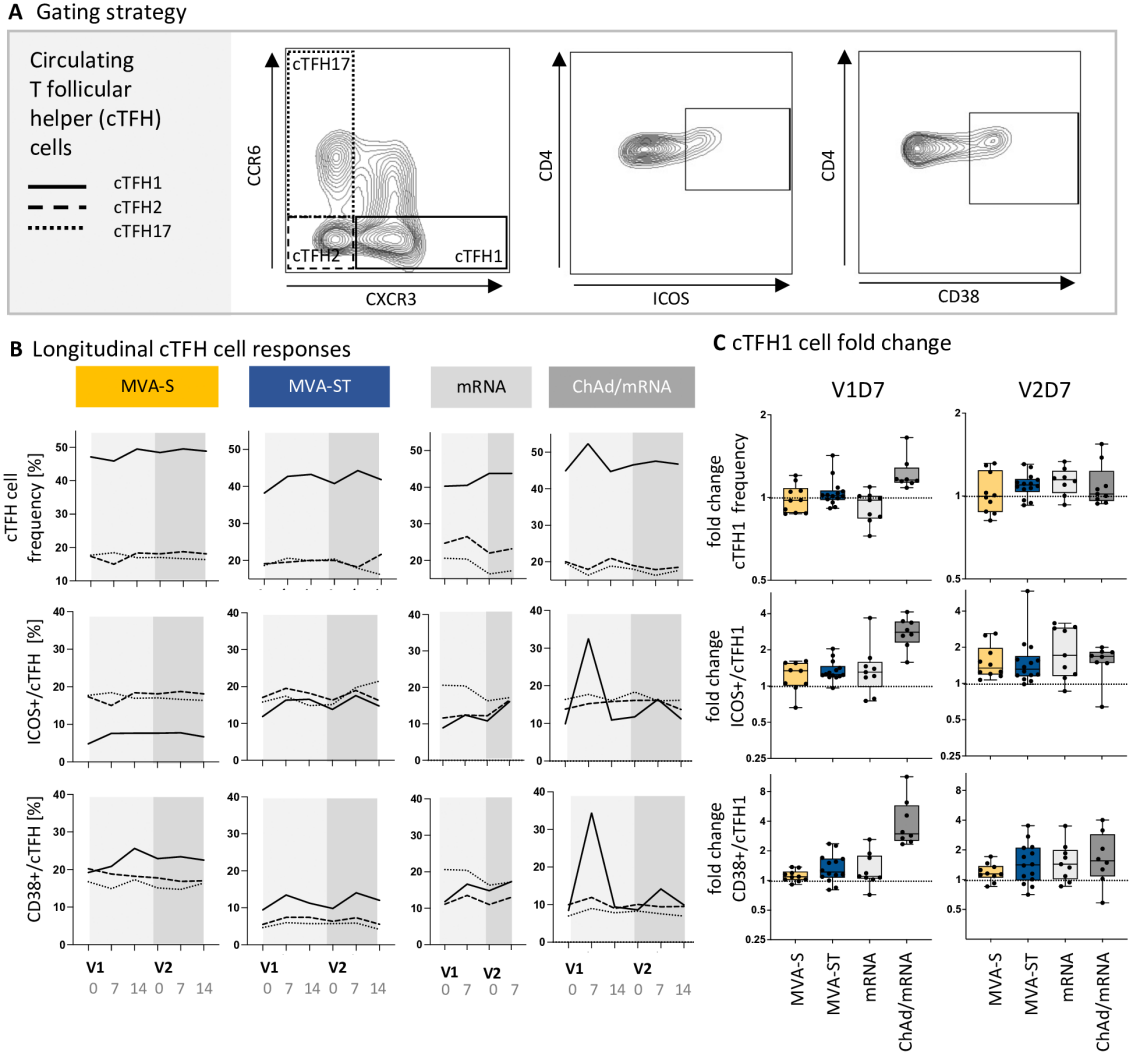
ChAd-based primary vaccination induces a stronger cTFH cell response compared to mRNA- and MVA-based vaccination. **(A)** Exemplary contour plots used for identification of cTFH cell subsets (cTFH1, cTFH2, cTFH17) and their activation status (based on CD38 and ICOS expression). The complete gating strategy for identification of cTFH cells within PBMCs is shown in **Supplementary Figure 2**. **(B)** Longitudinal dynamics of cTFH subpopulation frequencies, as well as ICOS+ and CD38+ cTFH cells, represented as median values of the subpopulations cTFH1, cTFH2 and cTFH17 on D0, D7 and D14 after V1 and V2. **(C)** Fold induction of cTFH1 cell frequency (upper panel), ICOS+ (middle panel) and CD38+ cTFH1 cells (lower panel) on V1D7 and V2D7 compared to baseline. Descriptive statistics for all parameters are reported in **Supplementary Tables 22**-**30**.

Differential stimuli of the innate immune response may drive the polarization of the TFH response, which in turn impacts B cell activation, differentiation, and antibody responses. We therefore investigated whether cTFH cells influence the distinct immunogenicity outcomes of MVA-S and MVA-ST vaccination. cTFH cell responses were measured based on PBMCs collected at D0, D7 and D14 after each vaccination, following restimulation with overlapping peptide pools M1-M4, spanning the SARS-CoV-2 spike protein. The analysis included different cTFH cell subsets (cTFH1, cTFH2, cTFH17) and activation markers (ICOS, CD38) (**Figures 4A, B**). We detected a strong cTFH cell response after the first vaccination with ChAd. We further characterized this response as a cTFH1 cell response (mfc ICOS = 2.8, CD38 = 3.0), whereas no increase in frequency and activation of cTFH2 or cTFH17 cells was observed (mfc < 1.1). A single mRNA vaccination only led to a slight increase in the subset of activated cTFH1 cells in some study participants (mfc ICOS = 1.3, mfc CD38 = 1.1). Similarly, a single vaccination with MVA-S or MVA-ST only induced a small increase in activated cTFH1 cells (mfc MVA-S, ICOS = 1.3, MVA-S, CD38 = 1.1, MVA-ST, ICOS = 1.3, MVA-ST, CD38 = 1.2) (**Figures 4B, C**). Following V2 of the MVA-S, MVA-ST, mRNA and ChAd/mRNA cohort, the frequency of activated (ICOS- or CD38-expressing) cTFH1 cells was slightly increased compared to baseline (mfc MVA-S, ICOS = 1.3, MVA-S, CD38 = 1.1, MVA-ST, ICOS = 1.3, MVA-ST, CD38 = 1.4, mfc mRNA, ICOS = 1.7, mRNA, CD38 = 1.4, ChAd/mRNA, ICOS = 1.7, ChAd/mRNA, CD38 = 1.6) (**Figure 4C**).

### 3.5 Increased immunogenicity of MVA-ST correlates with transcriptional, monocyte and cTFH cell responses

**Figure 5:**
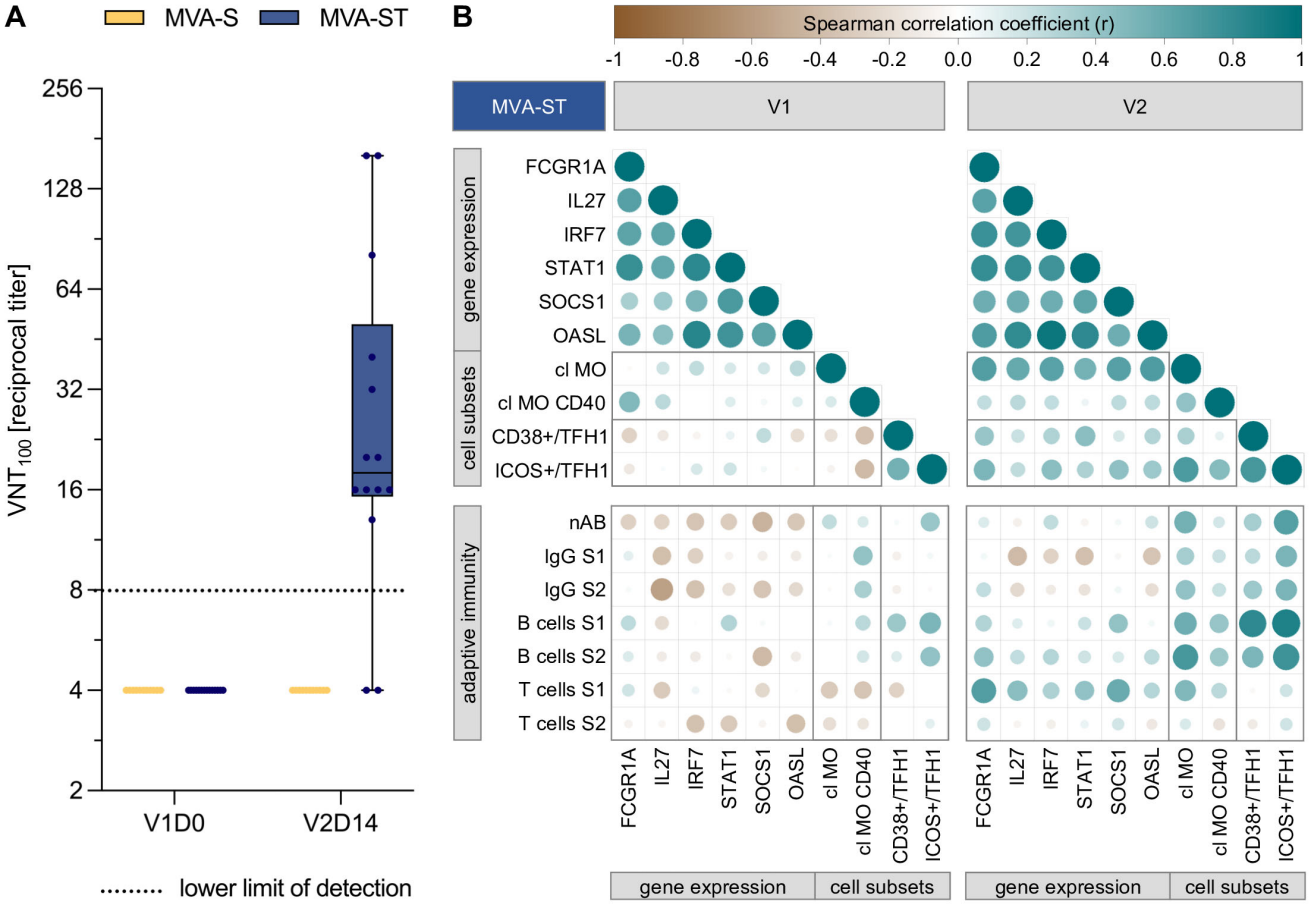
Increased immunogenicity of MVA-ST correlates with transcriptional, monocyte and cTFH cell responses. **(A)** Neutralizing antibodies (nAB), as measured by virus neutralization test (VNT_100_) against wild type SARS-CoV- 2 at baseline and 14 days after second vaccination with MVA-S and MVA-ST **(B)** Spearman correlation analysis between early immune responses following first (left panel) and second vaccination (right panel) with MVA-ST, and adaptive immune outcomes after completion of the vaccination schedule. The early immune parameters include the peak time point of previously selected DEGs, as well as the frequency and activation of classical monocytes (cl MO) and circulating T follicular helper 1 (cTFH1) cells. The adaptive immune parameters include nAB, S1- and S2-specific IgG, B cell and T cell responses, measuered at V2D14. All parameters were normalized to their baseline levels (V1D0), using fold changes for early immune responses and baseline subtraction for adaptive immune parameters. Circle size and color indicate Spearman correlation coefficient (r).

Neutralizing antibodies, IgG and IgA against the spike protein as well as long-lasting antigen-specific B and T cell responses are crucial for protection against SARS-CoV-2 infection and disease (30–32). Vaccination with MVA-ST, encoding the prefusion-stabilized spike protein, induced increased neutralizing antibody titers compared to MVA-S, encoding the native spike protein (**Figure 5A**). Furthermore, we previously reported that vaccination with MVA-ST leads to an increased antibody, B and T cell response against the S1 subunit of the SARS-CoV-2 spike protein (11). To date, the underlying mechanisms of the differential immunogenicity outcomes following vaccination with the two MVA-based vaccines, encoding different conformations of the spike protein, are not fully understood.

In order to better understand which genes and early immune signatures are potential mediators of the increased immunogenicity of MVA-ST, we analyzed correlations of transcriptional, innate and cTFH cell responses with the neutralizing antibody responses, as well as S1/S2-specific IgG, B and T cell responses (**Figure 5B**). The correlogram includes the adaptive immune parameters after completion of the vaccination series (V2D14), and the peak time point of the previously selected DEGs (FCGR1A, STAT1, OASL, IL27, IRF7 and SOCS1), as well as the classical monocyte and cTFH1 cell frequency and activation, following the first (left panel) and second vaccination (right panel).

Following the first vaccination, the selected DEGs correlated moderately to strongly with each other (r = 0.34 to 0.84). A positive correlation was also observed between the gene expression of FCGR1A and the CD40 expression of classical monocytes (r = 0.48), while moderate negative correlations were identified between a number of DEGs and adaptive immune outcomes (IL27 vs. S2-specific IgG: r = -0.56; SOCS1 vs. nAB: r = -0.45; SOCS1 vs. S2-specific B cells: r = -0.42). cTFH1 cell activation positively correlated with spike-specific B cells (ICOS/cTFH1vs. S1-specific B cells: r = 0.51; ICOS/cTFH1vs. S2-specific B cells: r = 0.43).

Following the second vaccination, moderate to strong positive correlations (r = 0.54 to 0.94) were observed between all selected DEGs, as well as between the DEGs and the frequency of classical monocytes. A number of DEGs also correlated with activated CD38+ or ICOS+ cTFH1 cells (FCGR1A vs. CD38+/cTFH1: r = 0.41; STAT1 vs. CD38+/cTFH1: r = 0.45; FCGR1A vs. ICOS+/cTFH1: r = 0.51; IRF7 vs. ICOS+/cTFH1: r = 0.45; SOCS1 vs. ICOS+/cTFH1: r = 0.43). Furthermore, the frequency and activation of classical monocytes correlated with ICOS+ cTFH1 cells (r = 0.7 and 0.46, respectively). In turn, CD38+ and ICOS+ cTFH1 cells strongly correlated with S1-specific B cells (r = 0.82 and 0.87, respectively) and to a lower extend with S2-specific B cells (r = 0.5 and 0.76, respectively). ICOS+ cTFH1 cells also correlated with neutralizing antibodies (r = 0.67) and S1/S2-specific IgG (r = 0.51). In addition, the frequency of classical monocytes correlated moderately to strongly with neutralizing antibodies (r = 0.54), S2-specific IgG (r = 0.43), S1/S2-specific B cells (r = 0.56 and 0.71, respectively) as well as S1-specific T cells (r = 0.49). Finally, the expression of FCGR1A, IL27, STAT1, SOCS1 moderately correlated with S1-specific T cells (r = 0.68, 0.45, 0.42 and 0.59, respectively). SOCS1 gene expression correlated moderately with S1-, and FCGR1A gene expression with S2-specific B cells (r = 0.41 and 0.43, respectively). Correlations with r < |0.4| are not further discussed in this section.

## 4 Discussion

Vaccine platforms such as viral vectors and mRNA lipid nanoparticles enable the accelerated development of vaccines against emerging pathogens and were crucial for the rapid availability of vaccines against SARS-CoV-2 (1). However, the impact of different vaccine platforms on immunogenicity and potential adverse events are not fully understood and comprehensive side-by-side analyses of different vaccine platforms are scarce (29). Apart from the immune response towards the viral vector and the viral vector’s ability to infect the host cell, the design of the insert can have a strong impact on the immunogenicity of a vaccine, since it is shaping the adaptive immune response against the respective antigen (25, 33).

We previously reported that the MVA-based vaccine candidate encoding the prefusion-stabilized spike protein (MVA-ST) was more immunogenic than MVA-S, which encodes the native spike protein. More precisely, both vaccines induced antibody, B and T cell responses towards the S2 subunit of the spike protein, but MVA-ST induced significantly higher responses specific to the S1 subunit (11). A similar finding has been reported for an Ad26 vector-based SARS-CoV-2 vaccine encoding a stabilized version of the spike protein compared to the native spike protein (34). Mechanistically, prefusion-stabilization leads to increased expression of the spike protein on the cell surface and reduces shedding of the cleaved S1 subunit (10, 35–37). The concept that prefusion stabilization of the spike protein leads to higher immunogenicity has also been demonstrated for the MERS-CoV spike protein (38, 39). While the S2 subunit is known to be more conserved among different coronavirus species, immune responses to the receptor binding domain (RBD)-containing S1 subunit are thought to be particularly important for protection against SARS-CoV-2, due to its crucial role for binding to the host cell receptor (40). In addition, we here report that MVA-ST induced higher titers of virus neutralizing antibodies. Consequently, we aimed at identifying transcriptional as well as innate and cTFH cell responses that may drive the differential immunogenicity outcomes. Our study represents a systems-level analysis of innate and adaptive immune responses induced by MVA-based SARS-CoV-2 vaccine candidates in humans, in side-by-side comparison to two different licensed vaccines based on ChAd and mRNA platforms. Based on bulk RNA sequencing and other omics approaches, systems vaccinology can improve the understanding of immune mechanisms and facilitate the identification of correlates of protection (41–43).

The impact of the vaccine platform on transcriptional responses has been previously shown in mouse studies comparing vaccination with different vectors but based on the same antigen insert (44). Furthermore, the comparison of gene expression signatures following immunization with vaccines against different pathogens, based on different technologies, also indicated distinct transcriptional signatures for different vaccine types (45, 46). One factor often considered to be an advantage of viral vector vaccines is their capability to activate innate immunity, mediated by the expression of diverse pathogen associated molecular patterns (PAMPs) that can be recognized by PRRs on innate immune cells (47). The induction of early innate immune responses has been reported for multiple viral vector vaccines, including MVA- and adenovirus-based vaccines, and has been shown to be able to enhance vaccine immunogenicity, replacing the necessity of an adjuvant (25, 48–50). More precisely, MVA-based vaccines have been described to induce signaling of the TLR2-TLR6-MyD88, MDA-5-IPS-1, NALP3 inflammasome and cGAS-STING pathways (51, 52). Similar effects were observed for mRNA vaccines and have been suggested to depend on type 1 interferon-dependent MDA5 signaling (53, 54).

Our pathway analysis revealed both similarities and differences between the transcriptional profiles of MVA-, ChAd- and mRNA-based vaccines, all targeting the SARS-CoV-2 spike protein. While pathways related e.g. to interferon signaling, antigen presentation, antiviral responses, and activation of PRRs were induced by vaccines based on all three platforms, the more immunogenic vaccine regimens ChAd/mRNA and mRNA/mRNA induced additional signaling pathways that were not activated following vaccination with the MVA-based vaccines, such as BCR signaling, PI3K signaling in B lymphocytes, crosstalk between DCs and natural killer cells. Differences between the platforms were also observed regarding the dynamics of the transcriptional responses; while MVA- and ChAd-based vaccination already induced a transcriptional activation following a single vaccination, activated pathways were not detected after the first but after the second mRNA vaccination. Similar findings have been reported in a study by Ryan et al. comparing the same ChAd- and mRNA-based vaccines against SARS-CoV-2 (29). A systems vaccinology study by Arunachalam et al. also identified a much higher transcriptional response following the second compared to the first mRNA vaccination (54). Besides the vaccine platform, the vaccine dose may have contributed to the differential immunogenicity: the ChAd-based Vaxzevria vaccine, which induced stronger responses on the level of gene expression, monocytes and cTFH cells, was administered at doses of 5 × 10^10^ viral particles, while the MVA-based vaccines were given at doses between 1 × 10^7^ and 1 × 10^8^ IU.

In line with other studies on MVA-based vaccines, both MVA-S and MVA-ST induced a type I interferon response early after vaccination, which is thought to be induced by the recognition of viral dsDNA (55–59). While the involvement of Toll-like receptor (TLR)- and PKR-dependent pathways in the induction of MVA-mediated interferon signaling have been controversially discussed (51, 60, 61), an involvement of the cGAS-STING pathway has been reported by Zhong et al. and Dai et al. (52, 55). Following MVA-ST vaccination, the activated pathways related to the sensing of viral DNA included the signaling of PRR, PKR, and cGAS-STING, while surprisingly none of these pathways were activated by MVA-S. Similarly, MVA-ST induced an activation of pathways related to antigen presentation, OAS antiviral response, and macrophage classical activation, which were not induced by MVA-S.

Notably, we here report differential transcriptomic activation early after vaccination with MVA-S and MVA-ST, even though they are based on the same viral vector and differ only in their antigen insert, encoding different spike protein conformations. Activation of signaling pathways by MVA itself should be detectable following both MVA-S and MVA-ST vaccination. The finding that the pathways activation of IRF by cytosolic PRRs and recognition of bacteria and viruses by PRRs are only upregulated following vaccination with MVA-ST, but not MVA-S, is pointing towards a role of the prefusion-stabilized spike protein. Indeed, the recognition of the SARS-CoV-2 spike protein by PRRs such as C-type lectin receptors (CLRs) has been described by several studies and was suggested to be mediated by glycans attached to the RBD contained in the spike S1 subunit (62, 63). While the CLRs DC-SIGN, L-SIGN, and MGL were shown to interact with recombinant S1, neither DC-SIGN or L-SIGN bound to recombinant S2 (64). The differential role of the spike subunits for the recognition by innate immune receptors may thus contribute to the stronger transcriptional activation induced by MVA-ST vaccination in our study. *In vitro* experiments with MVA-S and MVA-ST revealed differential expression patterns of the spike S1 and S2 subunits as early as 16-18 hours post infection. Both vaccines induced an expression of S2 on the surface of Huh7 and A549 cells, while only MVA-ST induced a robust expression of S1 (10). One hypothesis, based on the observed induction of PRR signaling following MVA-ST but not MVA-S vaccination, is that the presented S1 protein subunit is recognized by PRRs.

Following the second vaccination with MVA-ST, we observed overall positive correlations between upregulated DEGs, the induction of classical monocytes, and the activation of cTFH1 cells. In turn, the induction of classical monocytes and activation of cTFH1 cells (and to a lesser extent also the DEGs) correlated positively with adaptive immune responses, especially the induction of spike-specific B cells and neutralizing antibodies. While MVA-S and MVA-ST induced similarly low responses of innate immune cells, we observed a slightly stronger induction of CD38-expressing activated cTFH1 cells following MVA-ST compared to MVA-S vaccination. Notably, this subset also showed one of the strongest positive correlations with S1-specific B cell responses induced by MVA-ST and may therefore be one of the parameters involved in the differential immune responses to the two vaccines. This hypothesis is also supported by the predicted upstream regulators identified for the gene expression profiles induced by MVA-ST but not MVA-S vaccination, which included IL21 and CD40L, two of the most important activation signals provided to B cells by TFH cells (65–67). Indeed, cTFH cells have been described to be crucial for the development of B cell immunity and antibody responses following vaccination (18, 19). Correlations between the induction of cTFH1 cells and humoral immune responses following vaccination and infection have been described by several studies, in line with our findings (18, 20, 22, 68, 69). Since the investigated cTFH cells only represent a surrogate of TFH cells in germinal centers, and previous studies have shown that cTFH cells are only present for a short time after vaccination, an evaluation of TFH cells from lymphoid tissue could give additional valuable insights (16, 70). To this end, fine-needle aspiration of draining axillary lymph nodes following vaccination, as applied by Mudd et al. in individuals vaccinated with the BNT162b2 mRNA vaccine, could give insights into TFH cell responses following vaccination with the MVA-based vaccines (70). While fine-needle aspiration is invasive and therefore not a routine approach in clinical trials, investigation of MVA-based vaccines in immune organoids could give more insights into the dynamics of TFH cell responses (71, 72).

Despite the beneficial effects of vector-specific immune responses, they may also have detrimental effects on vaccine-induced immunogenicity. For example, a systems immunology study by Ryan et al., comparing innate and adaptive immune responses towards mRNA and ChAd vaccines, found a memory-like response of cTFH cells directed against the adenoviral vector which correlated with the abundance of multiple coagulation and complement proteins in plasma and might therefore be linked to the rare but severe adverse event of vaccine-induced immune thrombotic thrombocytopenia (VITT) (29). Since these side effects are highly relevant for future vaccine design, future studies should evaluate whether vaccines based on other viral vectors such as MVA might elicit the same potentially harmful immune signature of an increased vector-specific cTFH cell response. While we did not analyze vector-specific cTFH cells in our study, we report on responses of insert-specific cTFH cells which are critical for vaccine efficacy (18, 19) and were found to be less pronounced after MVA-based compared to ChAd-based vaccination. Notably, both the ChAd/mRNA and mRNA/mRNA regimens, but not the MVA-based vaccine candidates, induced a transcriptional response of the complement cascade, which is also suggested to be involved in the pathogenesis of VITT (73). This is in line with the beneficial safety profile of recombinant and non-recombinant MVA-based vaccines reported in numerous studies (8, 74).

Taken together, our study highlights the effect of both the vaccine platform and antigen insert on vaccine-induced immune responses in humans. While the small sample size limits the generalizability of the findings, the key strengths of our study include the frequent and longitudinal sampling of each study participant, as well as the analysis on both transcriptional and cellular levels. Our data reveal distinct early transcriptional signatures induced by two MVA-based vaccines encoding the native or prefusion-stabilized spike protein, indicating an impact of the antigen insert on early immune responses. An additional analysis of innate plasma cytokines would be of interest to validate these findings on the protein level. Positive correlations of classical monocyte and cTFH1 cell responses with neutralizing antibody and B cell responses following MVA-ST vaccination provide insight into potential mechanisms involved in the superior immunogenicity of MVA-ST compared to MVA-S. We believe that stabilization of the antigen insert might be beneficial in future vector vaccine design. Comparing the MVA-based vaccine candidates to licensed ChAd and mRNA vaccination regimens, we found that ChAd-based primary vaccination induces stronger transcriptional activation, classical monocyte and cTFH cell responses. While underlining the impact of the vaccine platform, the differential immunogenicity observed in our study may also be partially caused by the different dosages of the vaccines. Collectively, our findings may contribute to a better understanding of the influence of both vaccine platform and antigen insert on immunogenicity, which may be beneficial for future vaccine design.

## Data Availability

The original contributions presented in the study are publicly available. The RNA sequencing datasets for this study can be found in Gene expression omnibus (GEO, accession number GSE276544). Flow cytometry data can be found in Dryad (doi:10.5061/dryad.rjdfn2zmw).

## MVA-SARS-2 Study Group

The members of the MVA-SARS-2 Study Group are Amelie Alberti, Stefanie Gräfe, Cordula Grüttner, Svenja Hardtke, Jana Kochmann, Niclas Renevier, Maren Sandkuhl, Claudia Schlesner and Yashin Simsek (affiliated with the Institute for Infection Research and Vaccine Development (IIRVD), Center for Internal Medicine, University Medical Center Hamburg-Eppendorf), as well as Oliver A. Cornely, Tatjana Lammertz, Rosanne Sprute and Androniki Kolovou (affiliated with the Institute of Translational Research, CECAD, and the Department I of Internal Medicine, Faculty of Medicine and University Hospital Cologne, University of Cologne).
