# Supplementary material for: MVA-based SARS-CoV-2 vaccine candidates encoding different spike protein conformations induce distinct early transcriptional responses which may impact subsequent adaptive immunity
Source: Front Immunol. 2024 Dec 19;15:1500615. doi: 10.3389/fimmu.2024.1500615 (PMC11693667; doi:10.3389/fimmu.2024.1500615)
Supplement: Supplementary file 1 [file DataSheet1.pdf]

Supplementary Material

1 Supplementary Figures

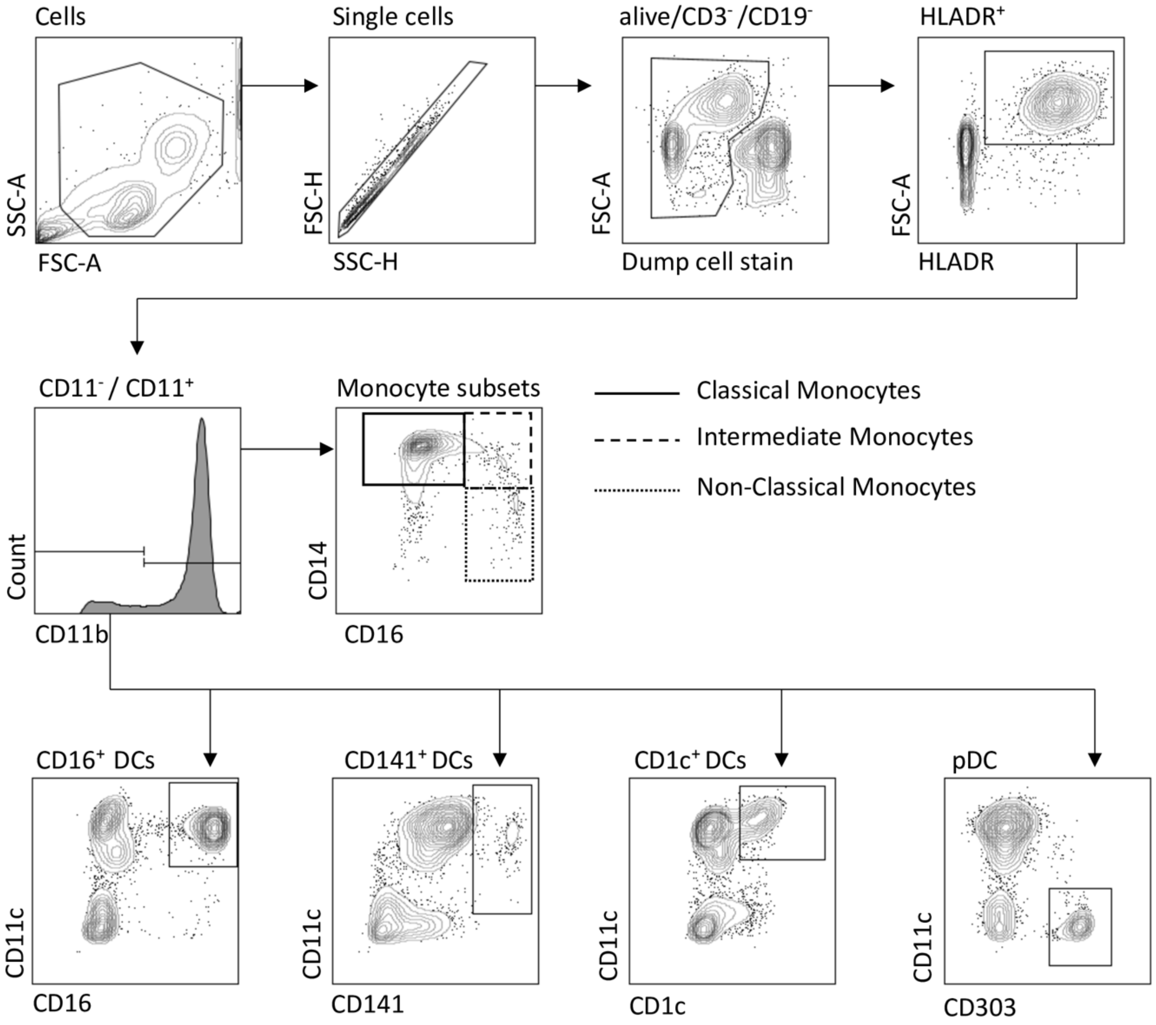

Supplementary Figure 1: Gating strategy for flowcytometric analysis of monocyte and dendritic cell subsets within PBMC. Cells were identified by their size and granularity using the forward scatter (FSC) and sideward scatter (SSC) signals. After doublets were excluded, dead cells, along with CD3+ and CD19+ cells, were removed ("dump" channel). Subsequently, HLA-DR+ cells were identified and divided into CD11b- and CD11b+ subsets. Monocyte subsets (classical monocytes, intermediate monocytes, non-classical monocytes) were identified based on CD14 and CD16 expression. Dendritic cell subsets were identified based on CD16, CD141, CD1c and CD303 expression.

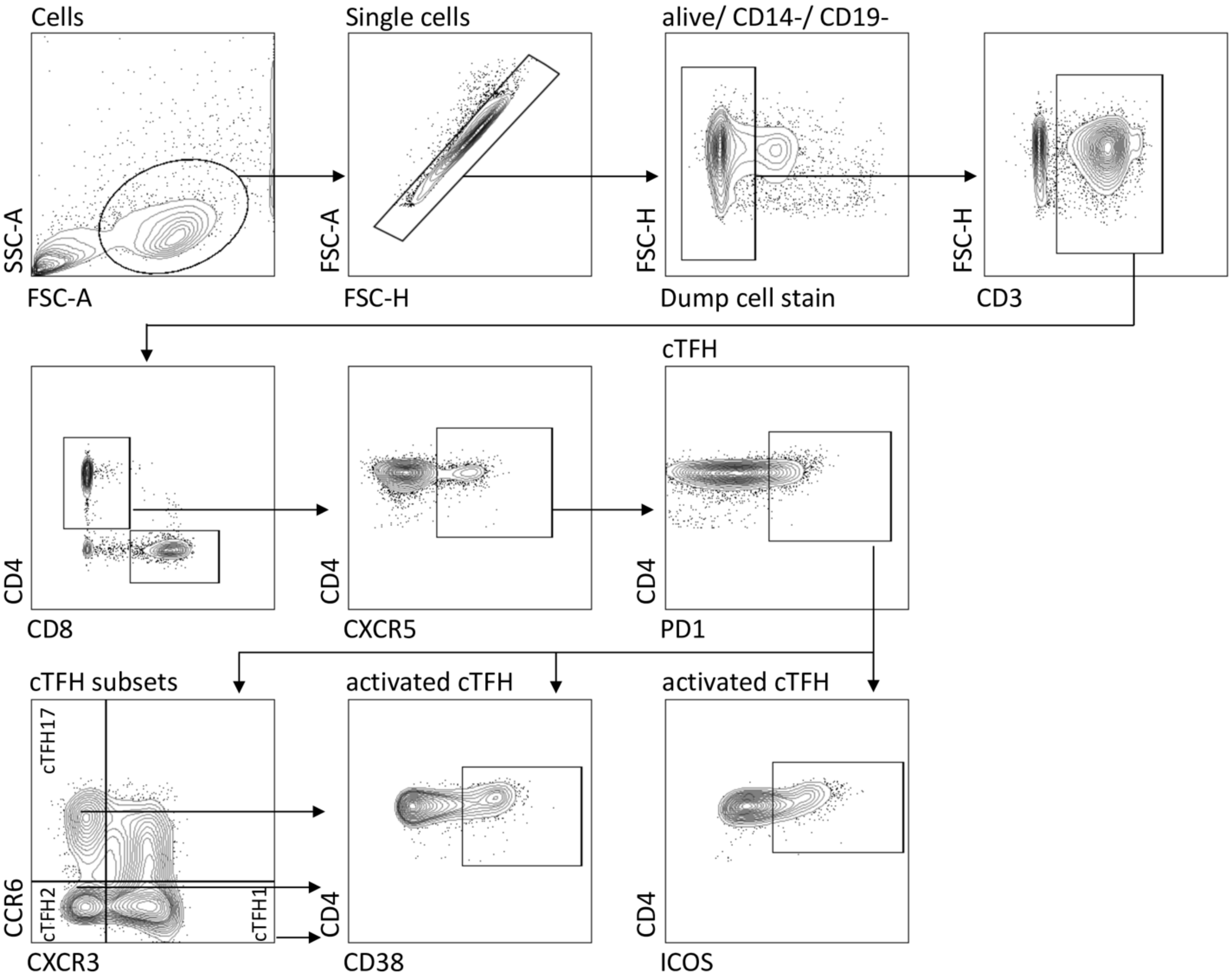

Supplementary Figure 2: Gating strategy for flowcytometric analysis of cTFH cells within PBMC. Cells were identified by their size and granularity using forward scatter (FSC) and sideward scatter (SSC) signals and in a next step, doublets were excluded. Subsequently, dead cells, CD14+ and CD19+ cells were excluded ("dump" channel). CD3+ cells were then identified and divided into CD4+ and CD8+ T cells. Within the CD4+ T cell population, circulating T follicular helper (cTFH) cells were identified based on CXCR5 and PD1 expression. cTFH subsets were identified based on CCR6 and CXCR3 expression and subsequently CD38 and ICOS expression was determined as activation marker.

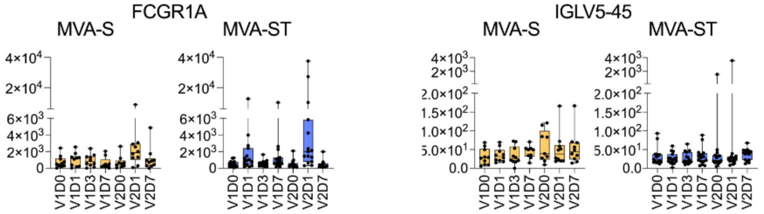

Supplementary Figure 3: Longitudinal expression of genes involved in the canonical signaling pathway *FCγR dependent phagocytosis*, which are differentially up- or downregulated on at least one time point following MVA-S or MVA-ST vaccination. Depicted are normalized counts calculated by variance stabilizing transformation (VST). Boxplots depict median and interquartile range, dots resemble individual data points.

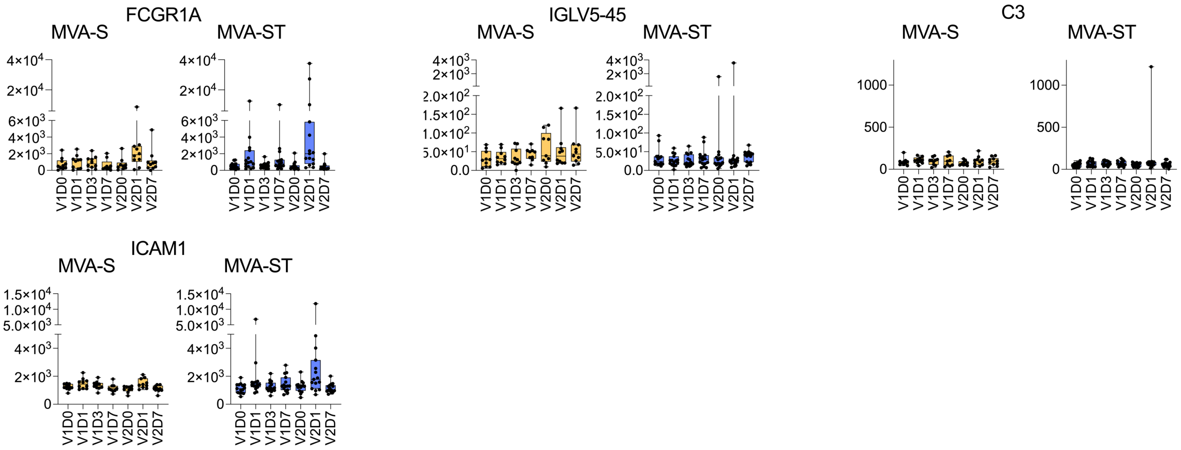

Supplementary Figure 4: Longitudinal expression of genes involved in the canonical signaling pathway *Interactions of lymphoid and non-lymphoid cells*, which are differentially up- or downregulated on at least one time point following MVA-S or MVA-ST vaccination. Depicted are normalized counts calculated by variance stabilizing transformation (VST). Boxplots depict median and interquartile range, dots resemble individual data points.

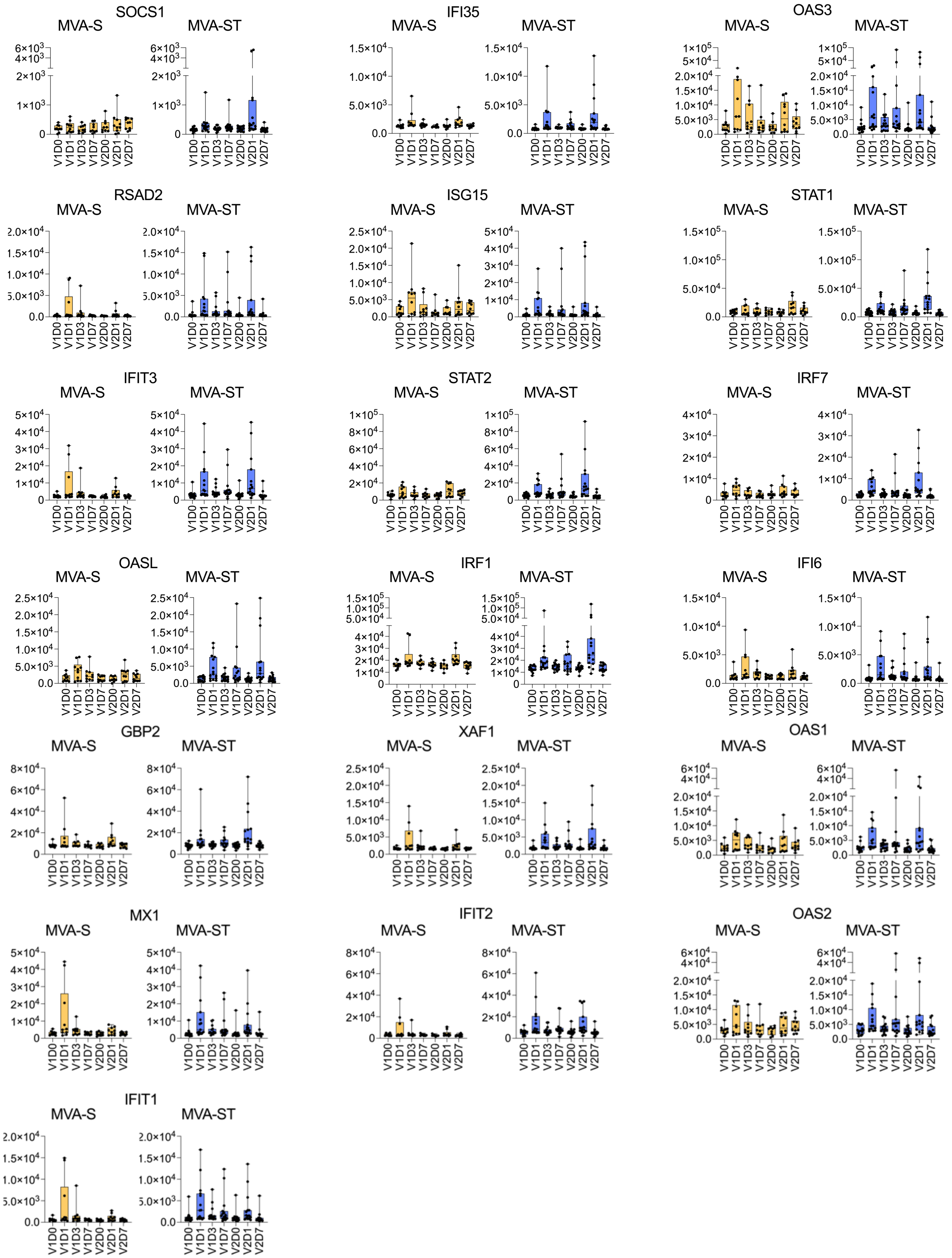

Supplementary Figure 5: Longitudinal expression of genes involved in the canonical signaling pathway *Interferon α/β signaling*, which are differentially up- or downregulated on at least one time point following MVA-S or MVA-ST vaccination. Depicted are normalized counts calculated by variance stabilizing transformation (VST). Boxplots depict median and interquartile range, dots resemble individual data points.

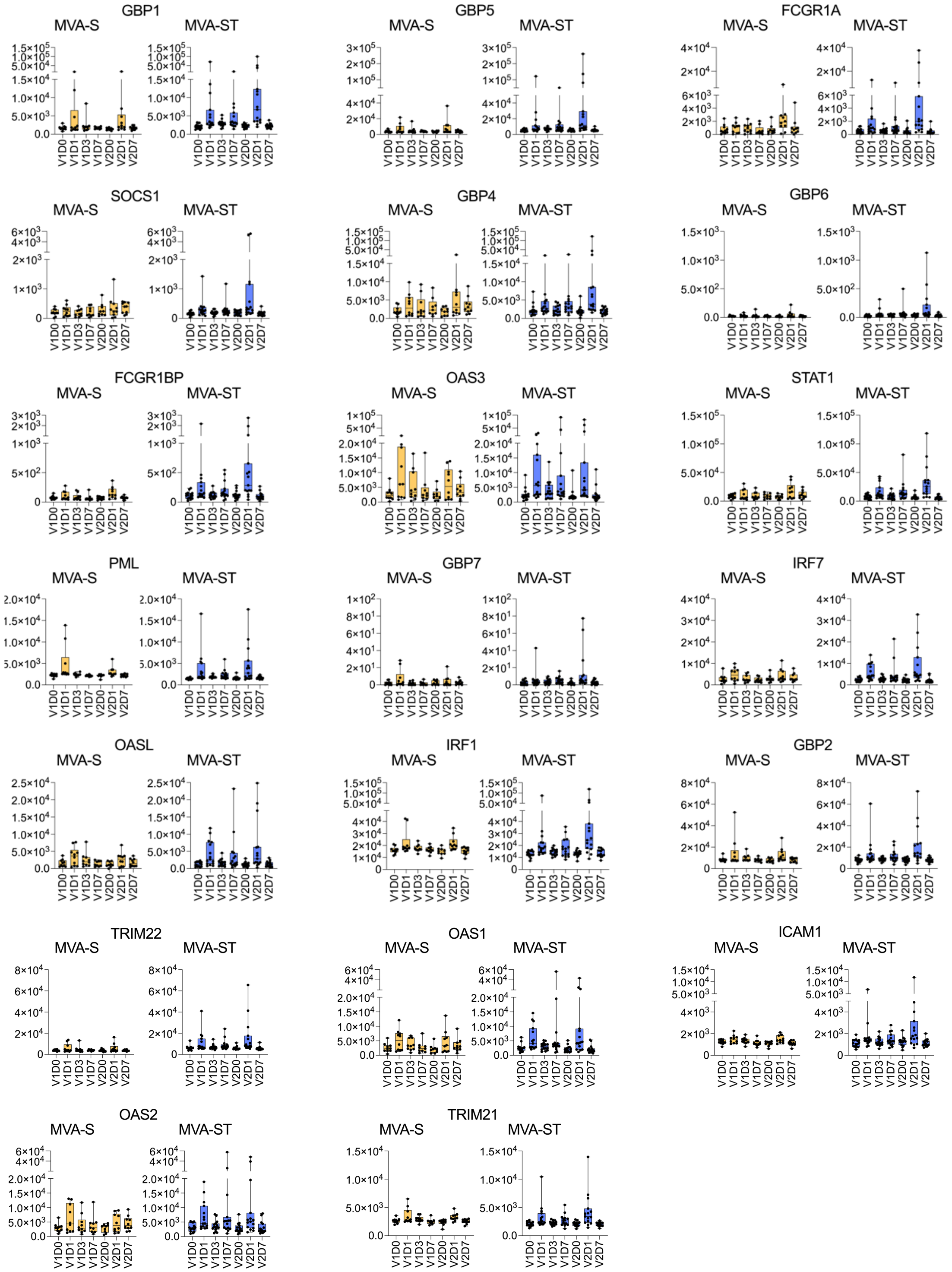

Supplementary Figure 6: Longitudinal expression of genes involved in the canonical signaling pathway *Interferon γ signaling*, which are differentially up- or downregulated on at least one time point following MVA-S or MVA-ST vaccination. Depicted are normalized counts calculated by variance stabilizing transformation (VST). Boxplots depict median and interquartile range, dots resemble individual data points.

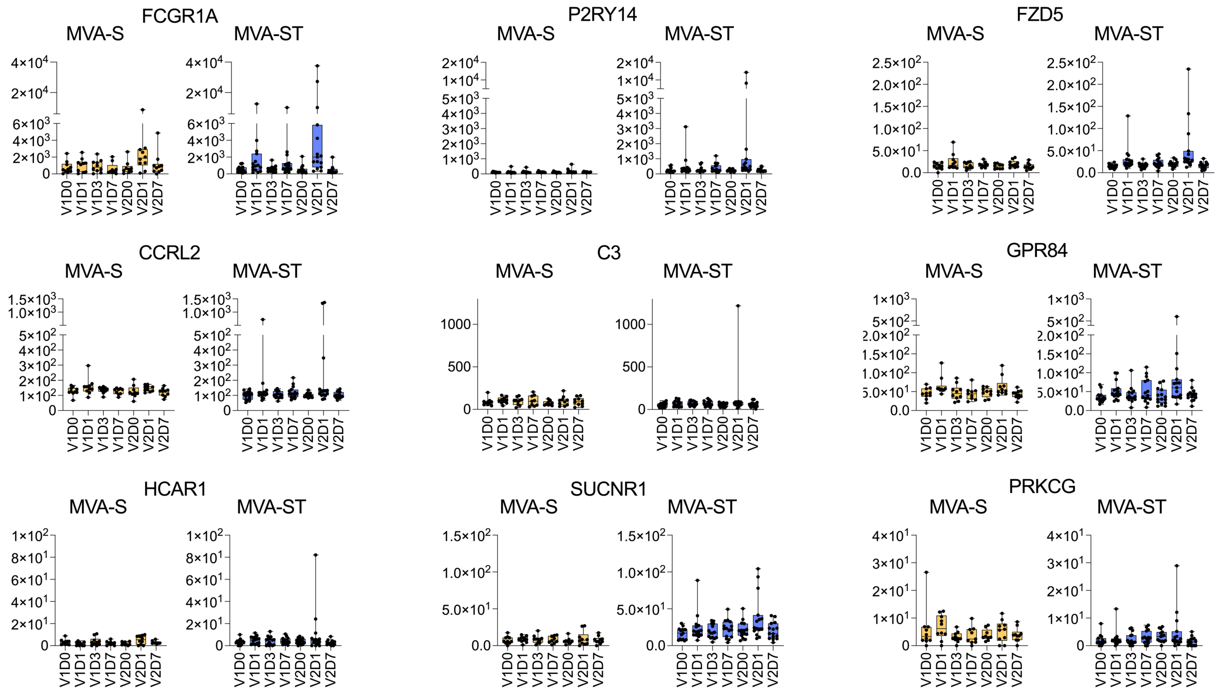

Supplementary Figure 7: Longitudinal expression of genes involved in the canonical signaling pathway *Phagosome formation*, which are differentially up- or downregulated on at least one time point following MVA-S or MVA-ST vaccination. Depicted are normalized counts calculated by variance stabilizing transformation (VST). Boxplots depict median and interquartile range, dots resemble individual data points.

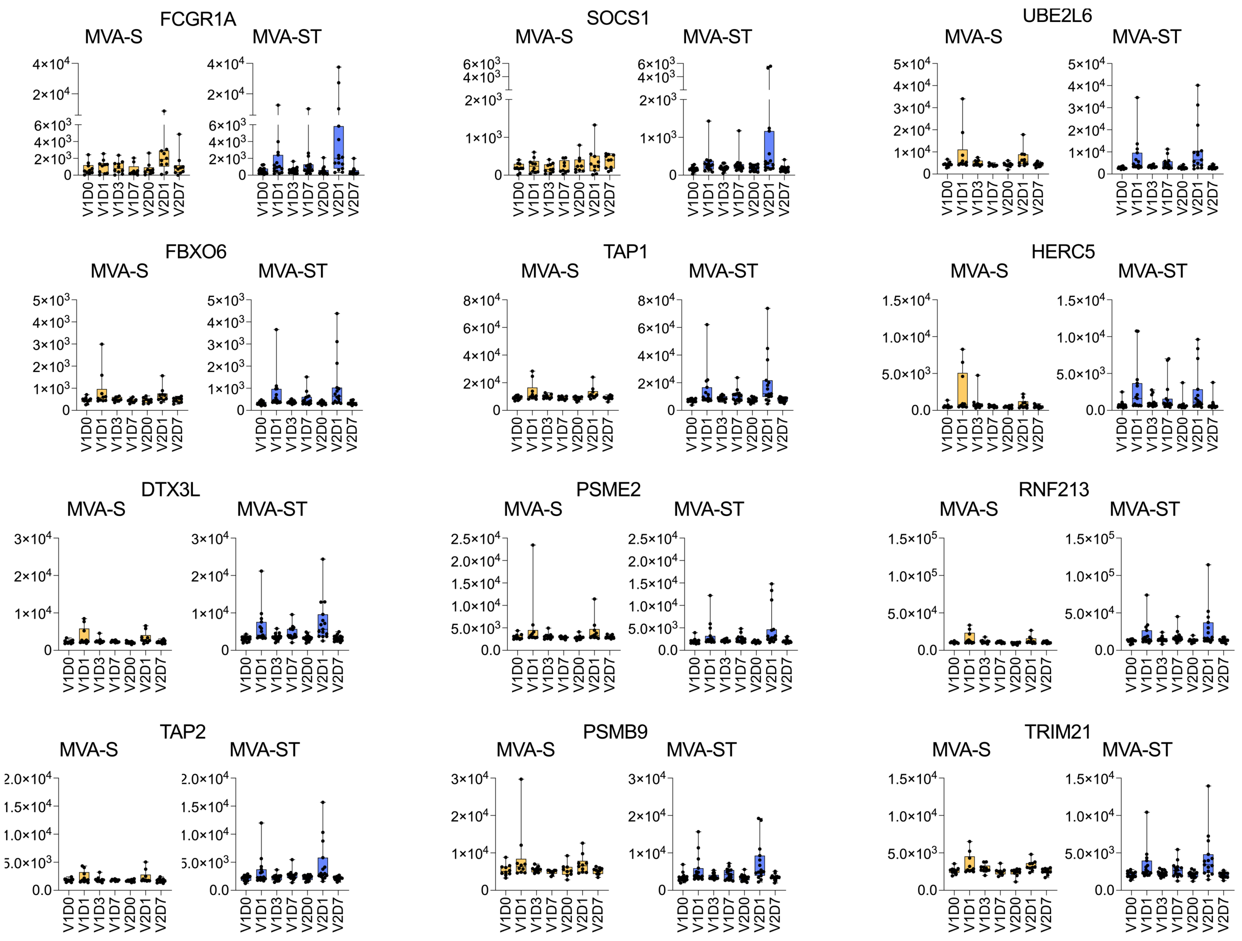

Supplementary Figure 8: Longitudinal expression of genes involved in the canonical signaling pathway *Class I mediated antigen presentation*, which are differentially up- or downregulated on at least one time point following MVA-S or MVA-ST vaccination. Depicted are normalized counts calculated by variance stabilizing transformation (VST). Boxplots depict median and interquartile range, dots resemble individual data points.

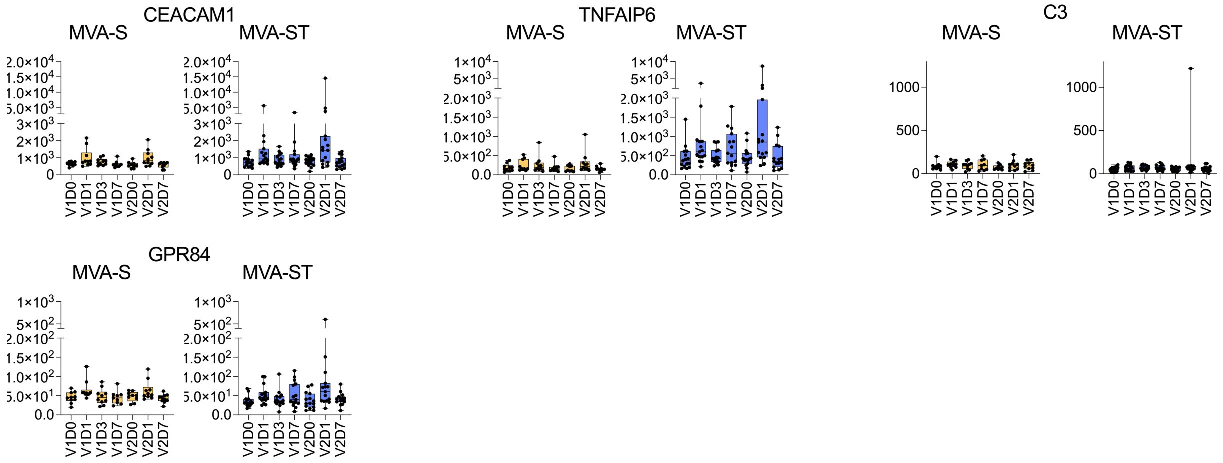

Supplementary Figure 9: Longitudinal expression of genes involved in the canonical signaling pathway *Neutrophil degranulation*, which are differentially up- or downregulated on at least one time point following MVA-S or MVA-ST vaccination. Depicted are normalized counts calculated by variance stabilizing transformation (VST). Boxplots depict median and interquartile range, dots resemble individual data points.

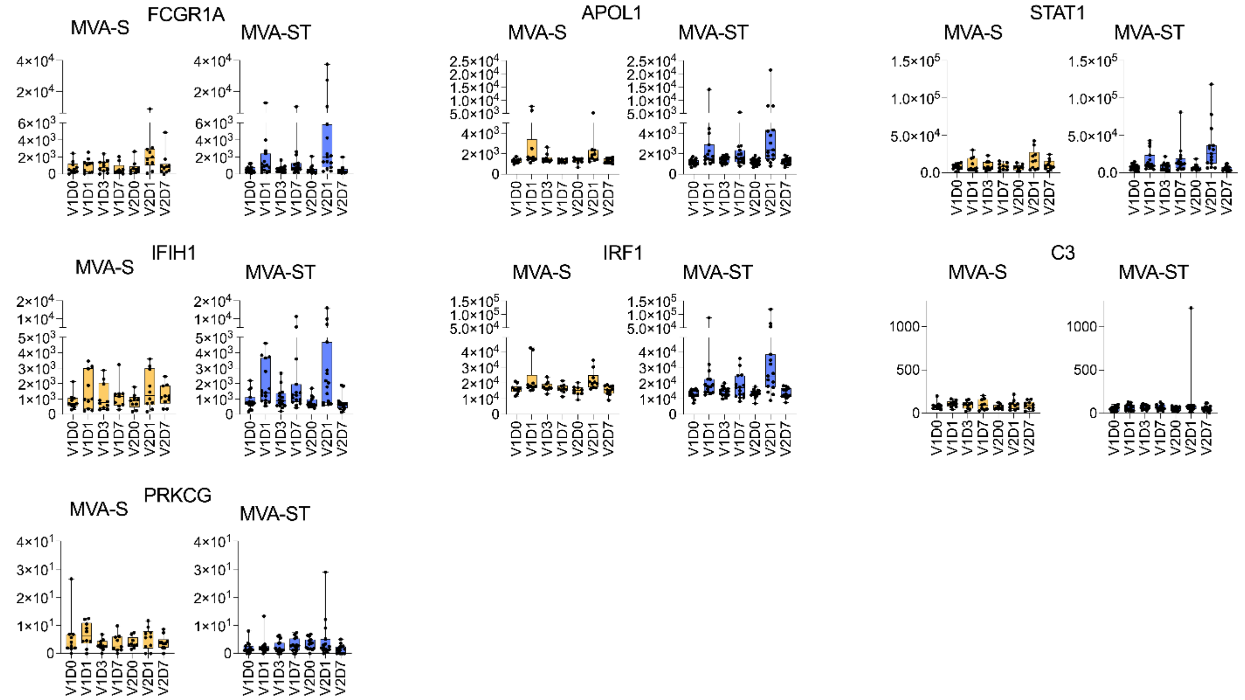

Supplementary Figure 10: Longitudinal expression of genes involved in the canonical signaling pathway *IL-12 signaling in macrophages*, which are differentially up- or downregulated on at least one time point following MVA-S or MVA-ST vaccination. Depicted are normalized counts calculated by variance stabilizing transformation (VST). Boxplots depict median and interquartile range, dots resemble individual data points.

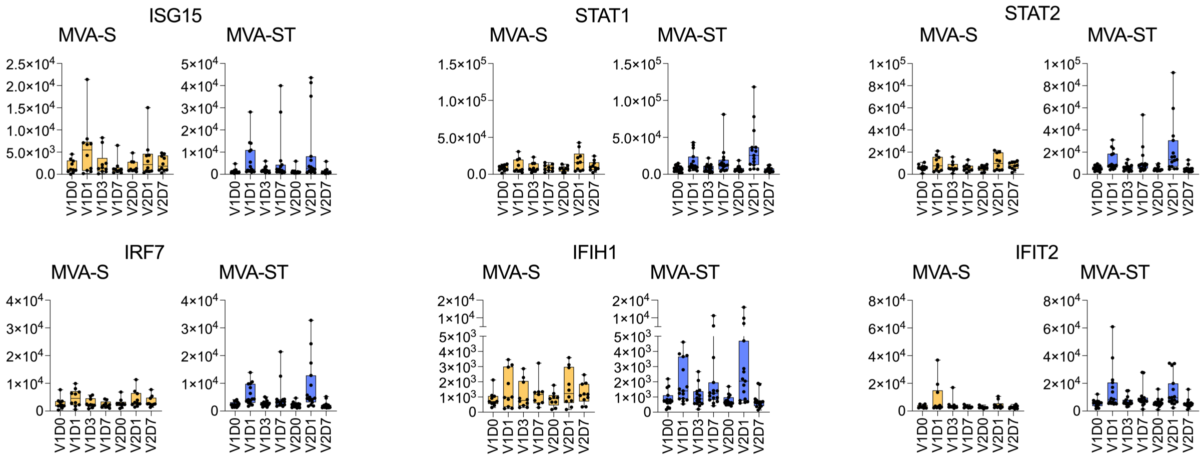

Supplementary Figure 11: Longitudinal expression of genes involved in the canonical signaling pathway *Activation of IRF by cytosolic pattern recognition receptors (PRR)*, which are differentially up- or downregulated on at least one time point following MVA-S or MVA-ST vaccination. Depicted are normalized counts calculated by variance stabilizing transformation (VST). Boxplots depict median and interquartile range, dots resemble individual data points.

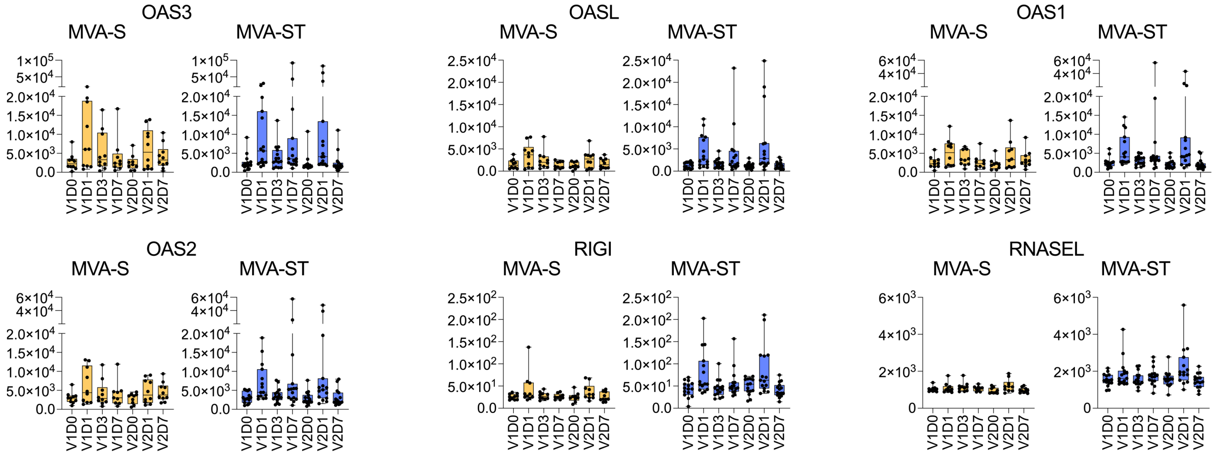

Supplementary Figure 12: Longitudinal expression of genes involved in the canonical signaling pathway *OAS antiviral response*, which are differentially up- or downregulated on at least one time point following MVA-S or MVA-ST vaccination. Depicted are normalized counts calculated by variance stabilizing transformation (VST). Boxplots depict median and interquartile range, dots resemble individual data points.

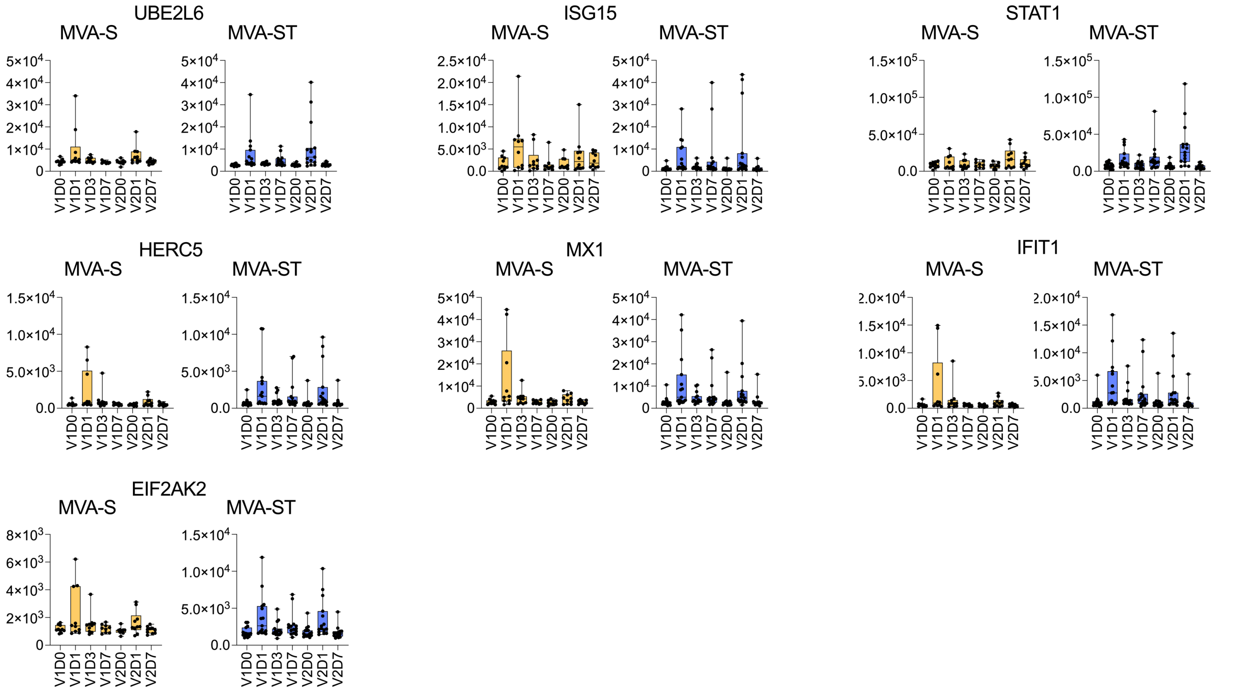

Supplementary Figure 13: Longitudinal expression of genes involved in the canonical signaling pathway *ISG15 antiviral mechanism*, which are differentially up- or downregulated on at least one time point following MVA-S or MVA-ST vaccination. Depicted are normalized counts calculated by variance stabilizing transformation (VST). Boxplots depict median and interquartile range, dots resemble individual data points.

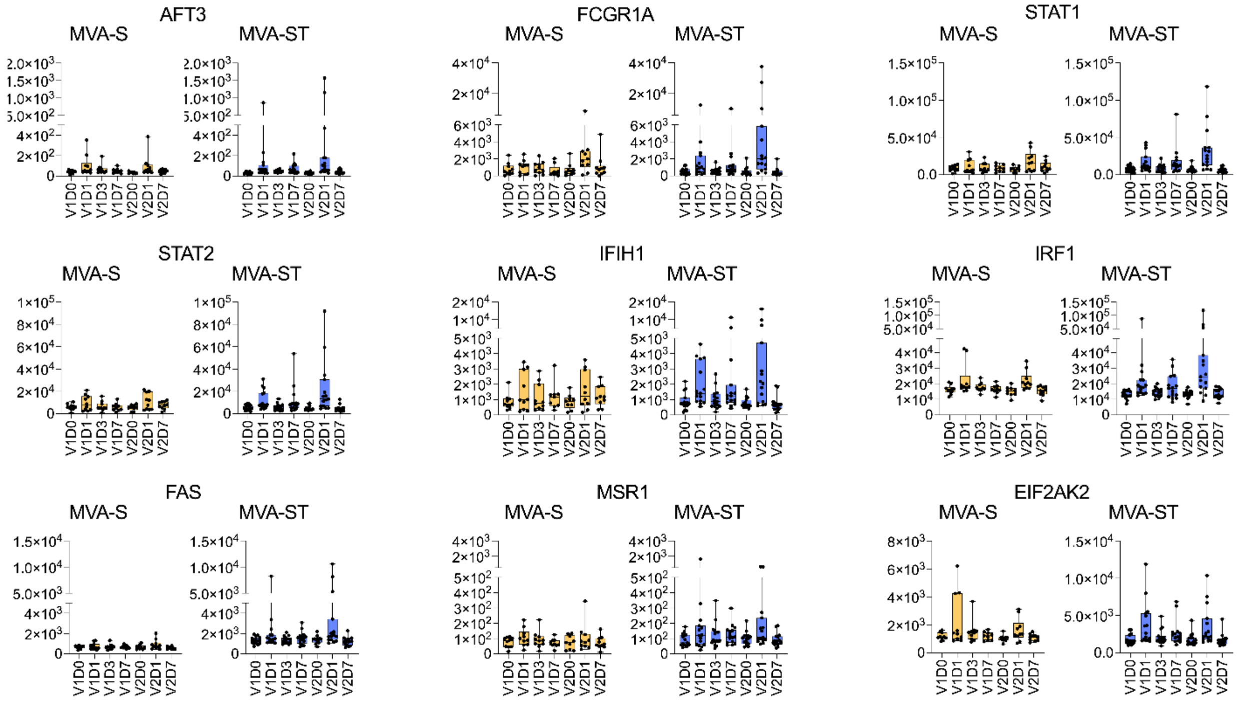

Supplementary Figure 14: Longitudinal expression of genes involved in the canonical signaling pathway *PKR in interferon induction and antiviral response*, which are differentially up- or downregulated on at least one time point following MVA-S or MVA-ST vaccination. Depicted are normalized counts calculated by variance stabilizing transformation (VST). Boxplots depict median and interquartile range, dots resemble individual data points.

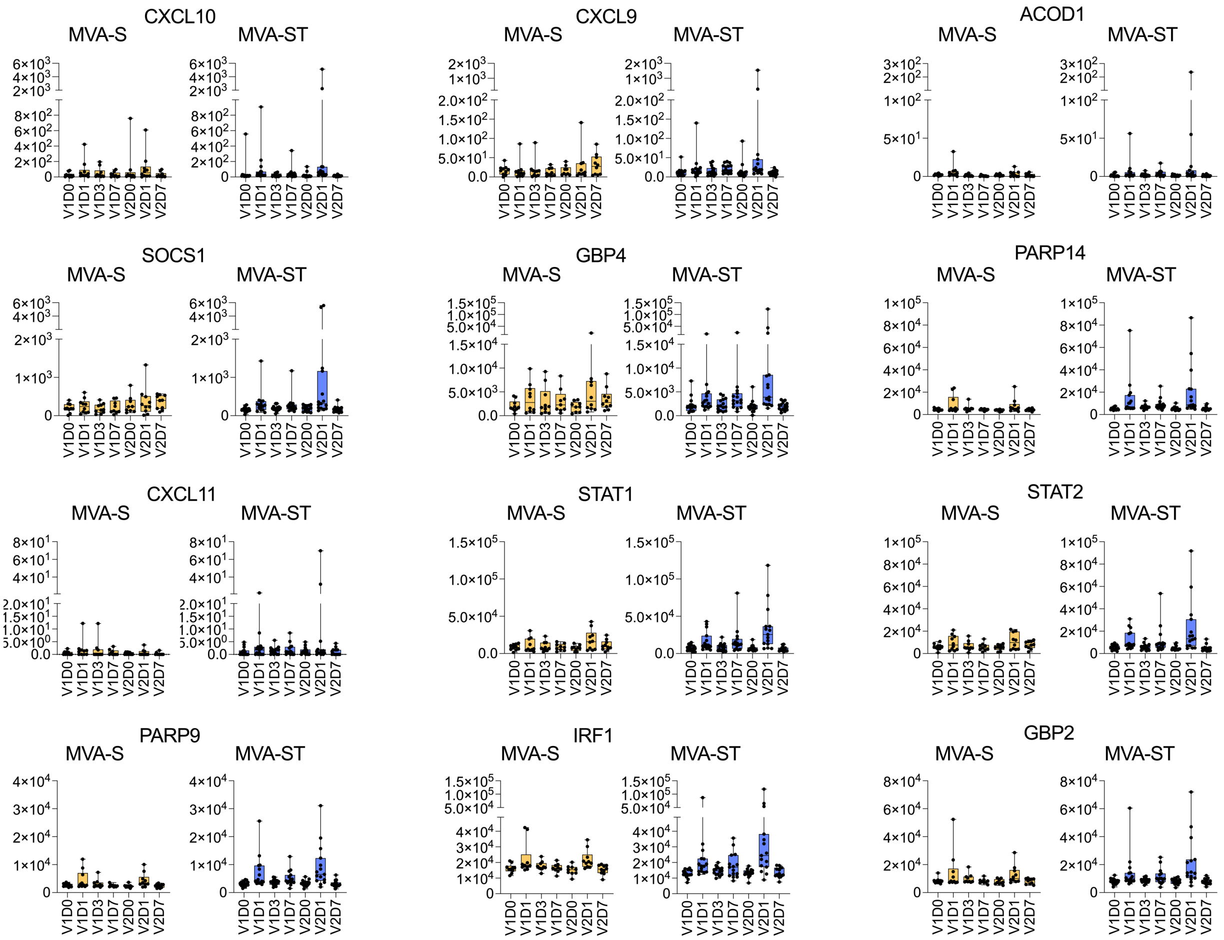

Supplementary Figure 15: Longitudinal expression of genes involved in the canonical signaling pathway *Macrophage classical activation signaling*, which are differentially up- or downregulated on at least one time point following MVA-S or MVA-ST vaccination. Depicted are normalized counts calculated by variance stabilizing transformation (VST). Boxplots depict median and interquartile range, dots resemble individual data points.

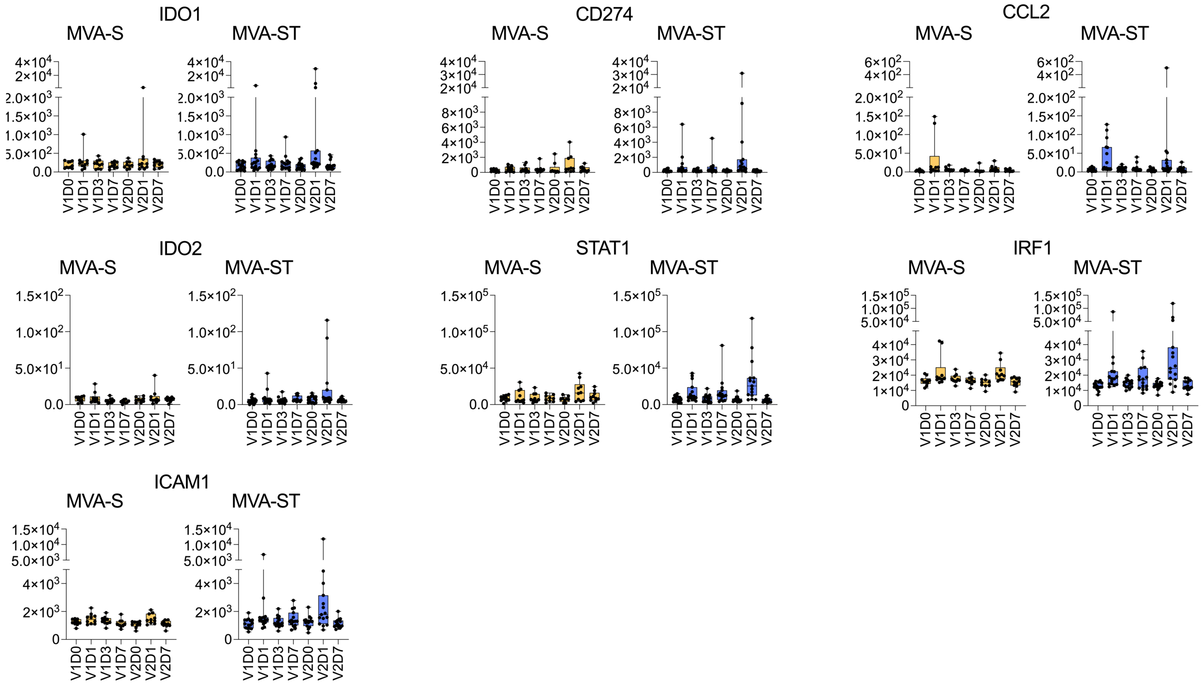

Supplementary Figure 16: Longitudinal expression of genes involved in the canonical signaling pathway *cGAS-STING pathway*, which are differentially up- or downregulated on at least one time point following MVA-S or MVA-ST vaccination. Depicted are normalized counts calculated by variance stabilizing transformation (VST). Boxplots depict median and interquartile range, dots resemble individual data points.

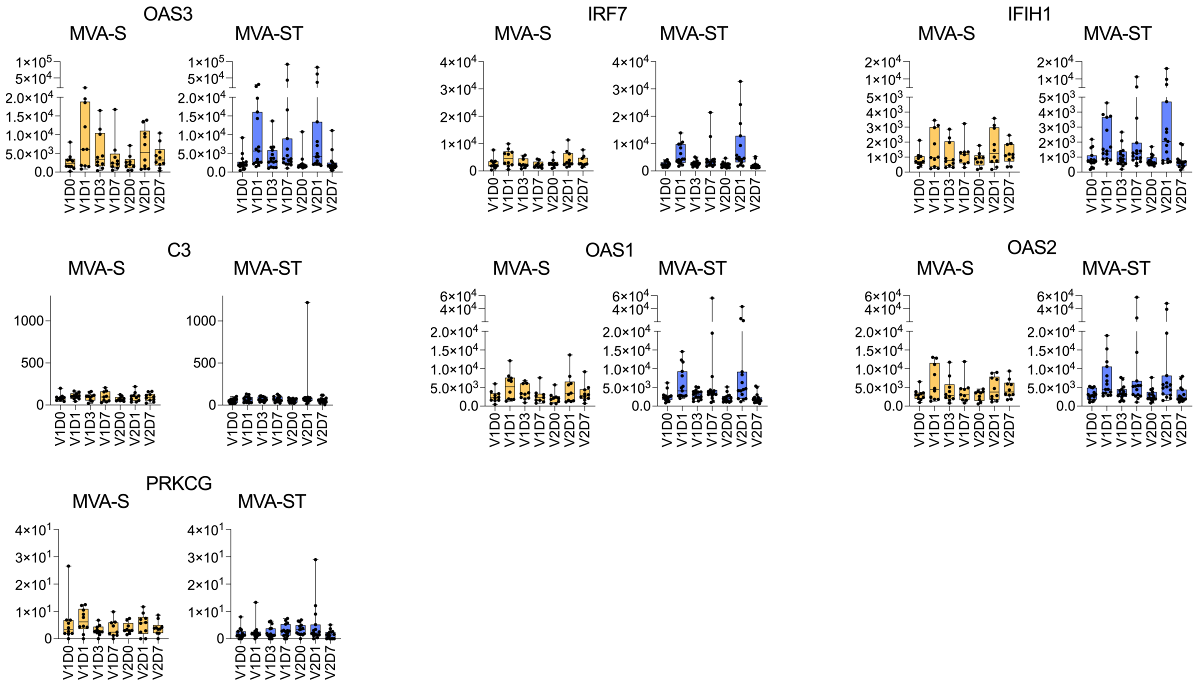

Supplementary Figure 17: Longitudinal expression of genes involved in the canonical signaling pathway *Recognition of bacteria and viruses by pattern recognition receptors (PRR)*, which are differentially up- or downregulated on at least one time point following MVA-S or MVA-ST vaccination. Depicted are normalized counts calculated by variance stabilizing transformation (VST). Boxplots depict median and interquartile range, dots resemble individual data points.

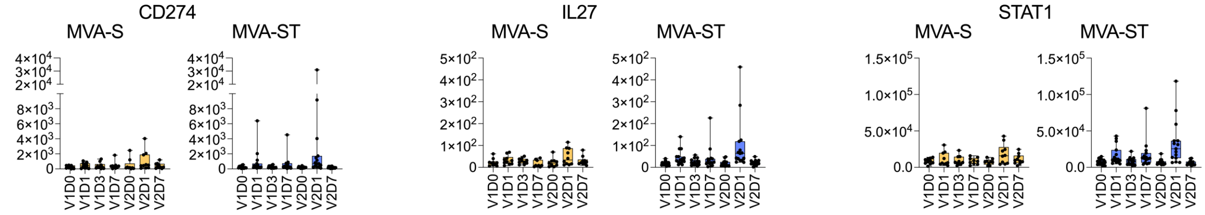

Supplementary Figure 18: Longitudinal expression of genes involved in the canonical signaling pathway *IL-27 signaling*, which are differentially up- or downregulated on at least one time point following MVA-S or MVA-ST vaccination. Depicted are normalized counts calculated by variance stabilizing transformation (VST). Boxplots depict median and interquartile range, dots resemble individual data points.

2 Supplementary Tables

Supplementary Table 1 – Baseline characteristics of study participants

	MVA-S N=12	MVA-ST N=15	mRNA N=10	ChAd/mRNA N=8
**Sex**				
female	**4 (33%)**	**10 (67%)**	**8 (80%)**	**9 (90%)**
male	**8 (67%)**	**5 (33%)**	**2 (20%)**	**1 (10%)**
**Age**				
median [years]	**40.5**	**41.0**	**31.5**	**31.5**
range [years]	**23 - 51**	**22 - 62**	**23 - 51**	**24 - 44**
**BMI**				
median [kg/m^2]	**26.2**	**22.7**	**21.8**	**21.2**
range [kg/m^2]	**18.6 – 28.8**	**19.1 – 29.8**	**18.3 – 25.2**	**20.5 – 24.6**
BMI=body-mass index.				

Supplementary Table 2 – Interval between vaccinations V1 and V2

	MVA-S	MVA-ST	mRNA	ChAd/mRNA
median (range) [days]	28	28	21 (21 – 23)	84 (64 – 84)

Supplementary Table 3 – Number of samples included into analysis of the MVA-S cohort

	V1D0	V1D1	V1D3	V1D7	V1D14	V2D0	V2D1	V2D3	V2D7	V2D14
**RNA Sequencing**	12	12	12	12	/	12	12	/	12	/
**Flow cytometry Innate cells**	10	10	10	10	/	10	10	/	10	/
**Flow cytometry cTFH cells**	10	/	/	10	10	10	/	/	10	10

Supplementary Table 4 – Number of samples included into analysis of the MVA-ST cohort

	V1D0	V1D1	V1D3	V1D7	V1D14	V2D0	V2D1	V2D3	V2D7	V2D14
**RNA Sequencing**	14	14	15	15	/	14	15	/	15	/
**Flow cytometry innate cells**	14	14	15	15	/	14	15	/	15	/
**Flow cytometry cTFH cells**	14	/	/	15	15	14	/	/	15	12

Supplementary Table 5 – Number of samples included into analysis of the mRNA cohort

	V1D0	V1D1	V1D3	V1D7	V1D14	V2D0	V2D1	V2D3	V2D7	V2D14
**RNA Sequencing**	8	10	10	10	/	10	10	10	10	/
**Flow cytometry innate cells**	9	9	9	9	/	9	9	9	9	/
**Flow cytometry cTFH cells**	9	/	/	9	/	9	/	/	9	/

Supplementary Table 6 – Number of samples included into analysis of the ChAd/mRNA cohort

	V1D0	V1D1	V1D3	V1D7	V1D14	V2D0	V2D1	V2D3	V2D7	V2D14
**RNA Sequencing**	8	8	8	8	/	6	8	6	8	/
**Flow cytometry innate cells**	8	8	8	8	/	6	8	7	8	/
**Flow cytometry cTFH cells**	8	/	/	8	7	6	/	/	8	8

Supplementary Table 7 – SARS-CoV-2 spike overlapping peptide pools

M1: aa 1-327	M2: aa 316-643	M3: aa 632-959	M4: aa 948-1273
MFVFLVLLPLVSSQC	NFRVQPTESIVRFPN	WRVYSTGSNVFQTRA	QDVVNQNAQALNTLV
LVLLPLVSSQCVNLT	QPTESIVRFPNITNL	STGSNVFQTRAGCLI	NQNAQALNTLVKQLS
PLVSSQCVNLTTRTQ	SIVRFPNITNLCPFG	NVFQTRAGCLIGAEH	QALNTLVKQLSSNFG
SQCVNLTTRTQLPPA	FPNITNLCPFGEVFN	TRAGCLIGAEHVNNS	TLVKQLSSNFGAISS
NLTTRTQLPPAYTNS	TNLCPFGEVFNATRF	CLIGAEHVNNSYECD	QLSSNFGAISSVLND
RTQLPPAYTNSFTRG	PFGEVFNATRFASVY	AEHVNNSYECDIPIG	NFGAISSVLNDILSR
PPAYTNSFTRGVYYP	VFNATRFASVYAWNR	NNSYECDIPIGAGIC	ISSVLNDILSRLDKV
TNSFTRGVYYPDKVF	TRFASVYAWNRKRIS	ECDIPIGAGICASYQ	LNDILSRLDKVEAEV
TRGVYYPDKVFRSSV	SVYAWNRKRISNCVA	PIGAGICASYQTQTN	LSRLDKVEAEVQIDR
YYPDKVFRSSVLHST	WNRKRISNCVADYSV	GICASYQTQTNSPRR	DKVEAEVQIDRLITG
KVFRSSVLHSTQDLF	RISNCVADYSVLYNS	SYQTQTNSPRRARSV	AEVQIDRLITGRLQS
SSVLHSTQDLFLPFF	CVADYSVLYNSASFS	QTNSPRRARSVASQS	IDRLITGRLQSLQTY
HSTQDLFLPFFSNVT	YSVLYNSASFSTFKC	PRRARSVASQSIIAY	ITGRLQSLQTYVTQQ
DLFLPFFSNVTWFHA	YNSASFSTFKCYGVS	RSVASQSIIAYTMSL	LQSLQTYVTQQLIRA
PFFSNVTWFHAIHVS	SFSTFKCYGVSPTKL	SQSIIAYTMSLGAEN	QTYVTQQLIRAAEIR
NVTWFHAIHVSGTNG	FKCYGVSPTKLNDLC	IAYTMSLGAENSVAY	TQQLIRAAEIRASAN
FHAIHVSGTNGTKRF	GVSPTKLNDLCFTNV	MSLGAENSVAYSNNS	IRAAEIRASANLAAT
HVSGTNGTKRFDNPV	TKLNDLCFTNVYADS	AENSVAYSNNSIAIP	EIRASANLAATKMSE
TNGTKRFDNPVLPFN	DLCFTNVYADSFVIR	VAYSNNSIAIPTNFT	SANLAATKMSECVLG
KRFDNPVLPFNDGVY	TNVYADSFVIRGDEV	NNSIAIPTNFTISVT	AATKMSECVLGQSKR
NPVLPFNDGVYFAST	ADSFVIRGDEVRQIA	AIPTNFTISVTTEIL	MSECVLGQSKRVDFC
PFNDGVYFASTEKSN	VIRGDEVRQIAPGQT	NFTISVTTEILPVSM	VLGQSKRVDFCGKGY
GVYFASTEKSNIIRG	DEVRQIAPGQTGKIA	SVTTEILPVSMTKTS	SKRVDFCGKGYHLMS
ASTEKSNIIRGWIFG	QIAPGQTGKIADYNY	EILPVSMTKTSVDCT	DFCGKGYHLMSFPQS
KSNIIRGWIFGTTLD	GQTGKIADYNYKLPD	VSMTKTSVDCTMYIC	KGYHLMSFPQSAPHG
IRGWIFGTTLDSKTQ	KIADYNYKLPDDFTG	KTSVDCTMYICGDST	LMSFPQSAPHGVVFL
IFGTTLDSKTQSLLI	YNYKLPDDFTGCVIA	DCTMYICGDSTECSN	PQSAPHGVVFLHVTY
TLDSKTQSLLIVNNA	LPDDFTGCVIAWNSN	YICGDSTECSNLLLQ	PHGVVFLHVTYVPAQ
KTQSLLIVNNATNVV	FTGCVIAWNSNNLDS	DSTECSNLLLQYGSF	VFLHVTYVPAQEKNF
LLIVNNATNVVIKVC	VIAWNSNNLDSKVGG	CSNLLLQYGSFCTQL	VTYVPAQEKNFTTAP
NNATNVVIKVCEFQF	NSNNLDSKVGGNYNY	LLQYGSFCTQLNRAL	PAQEKNFTTAPAICH
NVVIKVCEFQFCNDP	LDSKVGGNYNYLYRL	GSFCTQLNRALTGIA	KNFTTAPAICHDGKA
KVCEFQFCNDPFLGV	VGGNYNYLYRLFRKS	TQLNRALTGIAVEQD	TAPAICHDGKAHFPR
FQFCNDPFLGVYYHK	YNYLYRLFRKSNLKP	RALTGIAVEQDKNTQ	ICHDGKAHFPREGVF
NDPFLGVYYHKNNKS	YRLFRKSNLKPFERD	GIAVEQDKNTQEVFA	GKAHFPREGVFVSNG
LGVYYHKNNKSWMES	RKSNLKPFERDISTE	EQDKNTQEVFAQVKQ	FPREGVFVSNGTHWF
YHKNNKSWMESEFRV	LKPFERDISTEIYQA	NTQEVFAQVKQIYKT	GVFVSNGTHWFVTQR
NKSWMESEFRVYSSA	ERDISTEIYQAGSTP	VFAQVKQIYKTPPIK	SNGTHWFVTQRNFYE
MESEFRVYSSANNCT	STEIYQAGSTPCNGV	VKQIYKTPPIKDFGG	HWFVTQRNFYEPQII
FRVYSSANNCTFEYV	YQAGSTPCNGVEGFN	YKTPPIKDFGGFNFS	TQRNFYEPQIITTDN

SSANNCTFEYVSQPF	STPCNGVEGFNCYFP	PIKDFGGFNFSQILP	FYEPQIITTDNTFVS
NCTFEYVSQPFLMDL	NGVEGFNCYFPLQSY	FGGFNFSQILPDPSK	QIITTDNTFVSGNCD
EYVSQPFLMDLEGKQ	GFNCYFPLQSYGFQP	NFSQILPDPSKPSKR	TDNTFVSGNCDVVIG
QPFLMDLEGKQGNFK	YFPLQSYGFQPTNGV	ILPDPSKPSKRSFIE	FVSGNCDVVIGIVNN
MDLEGKQGNFKNLRE	QSYGFQPTNGVGYQP	PSKPSKRSFIEDLLF	NCDVVIGIVNNTVYD
GKQGNFKNLREFVFK	FQPTNGVGYQPYRVV	SKRSFIEDLLFNKVT	VIGIVNNTVYDPLQP
NFKNLREFVFKNIDG	NGVGYQPYRVVVLSF	FIEDLLFNKVTLADA	VNNTVYDPLQPELDS
LREFVFKNIDGYFKI	YQPYRVVVLSFELLH	LLFNKVTLADAGFIK	VYDPLQPELDSFKEE
VFKNIDGYFKIYSKH	RVVVLSFELLHAPAT	KVTLADAGFIKQYGD	LQPELDSFKEELDKY
IDGYFKIYSKHTPIN	LSFELLHAPATVCGP	ADAGFIKQYGDCLGD	LDSFKEELDKYFKNH
FKIYSKHTPINLVRD	LLHAPATVCGPKKST	FIKQYGDCLGDIAAR	KEELDKYFKNHTSPD
SKHTPINLVRDLPQG	PATVCGPKKSTNLVK	YGDCLGDIAARDLIC	DKYFKNHTSPDVDLG
PINLVRDLPQGFSAL	CGPKKSTNLVKNKCV	LGDIAARDLICAQKF	KNHTSPDVDLGDISG
VRDLPQGFSALEPLV	KSTNLVKNKCVNFNF	AARDLICAQKFNGLT	SPDVDLGDISGINAS
PQGFSALEPLVDLPI	LVKNKCVNFNFNGLT	LICAQKFNGLTVLPP	DLGDISGINASVVNI
SALEPLVDLPIGINI	KCVNFNFNGLTGTGV	QKFNGLTVLPPLLTD	ISGINASVVNIQKEI
PLVDLPIGINITRFQ	FNFNGLTGTGVLTES	GLTVLPPLLTDEMIA	NASVVNIQKEIDRLN
LPIGINITRFQTLLA	GLTGTGVLTESNKKF	LPPLLTDEMIAQYTS	VNIQKEIDRLNEVAK
INITRFQTLLALHRS	TGVLTESNKKFLPFQ	LTDEMIAQYTSALLA	KEIDRLNEVAKNLNE
RFQTLLALHRSYLTP	TESNKKFLPFQQFGR	MIAQYTSALLAGTIT	RLNEVAKNLNESLID
LLALHRSYLTPGDSS	KKFLPFQQFGRDIAD	YTSALLAGTITSGWT	VAKNLNESLIDLQEL
HRSYLTPGDSSSGWT	PFQQFGRDIADTTDA	LLAGTITSGWTFGAG	LNESLIDLQELGKYE
LTPGDSSSGWTAGAA	FGRDIADTTDAVRDP	TITSGWTFGAGAALQ	LIDLQELGKYEQYIK
DSSSGWTAGAAAYYV	IADTTDAVRDPQTLE	GWTFGAGAALQIPFA	QELGKYEQYIKWPWY
GWTAGAAAYYVGYLQ	TDAVRDPQTLEILDI	GAGAALQIPFAMQMA	KYEQYIKWPWYIWLG
GAAAYYVGYLQPRTF	RDPQTLEILDITPCS	ALQIPFAMQMAYRFN	YIKWPWYIWLGFIAG
YYVGYLQPRTFLLKY	TLEILDITPCSFGGV	PFAMQMAYRFNGIGV	PWYIWLGFIAGLIAI
YLQPRTFLLKYNENG	LDITPCSFGGVSVIT	QMAYRFNGIGVTQNV	WLGFIAGLIAIVMVT
RTFLLKYNENGTITD	PCSFGGVSVITPGTN	RFNGIGVTQNVLYEN	IAGLIAIVMVTIMLC
LKYNENGTITDAVDC	GGVSVITPGTNTSNQ	IGVTQNVLYENQKLI	IAIVMVTIMLCCMTS
ENGTITDAVDCALDP	VITPGTNTSNQVAVL	QNVLYENQKLIANQF	MVTIMLCCMTSCCSC
ITDAVDCALDPLSET	GTNTSNQVAVLYQDV	YENQKLIANQFNSAI	MLCCMTSCCSCLKGC
VDCALDPLSETKCTL	SNQVAVLYQDVNCTE	KLIANQFNSAIGKIQ	MTSCCSCLKGCCSCG
LDPLSETKCTLKSFT	AVLYQDVNCTEVPVA	NQFNSAIGKIQDSLS	CSCLKGCCSCGSCCK
SETKCTLKSFTVEKG	QDVNCTEVPVAIHAD	SAIGKIQDSLSSTAS	KGCCSCGSCCKFDED
CTLKSFTVEKGIYQT	CTEVPVAIHADQLTP	KIQDSLSSTASALGK	SCGSCCKFDEDDSEP
SFTVEKGIYQTSNFR	PVAIHADQLTPTWRV	SLSSTASALGKLQDV	CCKFDEDDSEPVLKG
EKGIYQTSNFRVQPT	HADQLTPTWRVYSTG	TASALGKLQDVVNQN	DEDDSEPVLKGVKLH
YQTSNFRVQPTESIV	LTPTWRVYSTGSNVF	LGKLQDVVNQNAQAL	DDSEPVLKGVKLHYT

Supplementary Table 8 – Descriptive statistics: frequency of classical monocytes at longitudinal time points following vaccination

Cohort	Statistical parameter	V1D0	V1D1	V1D3	V1D7	V2D0	V2D1	V2D3	V2D7
MVA-S	Sample size	10	10	10	10	10	10	0	10
	Minimum	4.84	8.11	5.20	2.82	5.03	7.80		2.81
	25% Percentile	9.42	10.70	7.10	9.22	8.63	10.70		8.86
	Median	13.65	14.00	14.55	11.55	13.55	14.65		12.35
	75% Percentile	17.68	18.35	18.33	16.35	16.10	18.20		15.60
	Maximum	18.60	20.20	21.40	19.30	19.00	24.80		25.40
MVA-ST	Sample size	14	14	15	15	14	15	0	15
	Minimum	6.46	4.59	4.35	4.16	5.24	6.65		4.17
	25% Percentile	7.93	8.69	7.18	7.50	8.18	8.75		7.48
	Median	9.55	10.30	8.65	10.50	11.90	13.90		11.40
	75% Percentile	12.10	14.05	12.40	11.60	13.43	16.40		12.10
	Maximum	18.20	21.70	18.90	19.50	17.10	24.20		16.80
mRNA	Sample size	9	9	9	9	9	9	8	9
	Minimum	4.42	4.78	3.50	4.04	4.63	9.12	5.84	4.66
	25% Percentile	7.88	6.84	6.89	8.74	8.28	12.10	9.83	5.48
	Median	10.10	10.30	9.07	9.31	9.71	18.90	11.75	8.21
	75% Percentile	11.80	13.75	12.35	11.10	12.50	25.75	13.60	11.15
	Maximum	15.40	18.00	13.90	14.20	18.40	28.80	16.50	13.40
ChAd/ mRNA	Sample size	8	8	8	8	6	8	7	8
	Minimum	5.34	9.84	7.08	6.52	7.58	8.78	8.69	5.44
	25% Percentile	7.87	11.28	7.23	7.21	7.88	10.80	8.71	6.06
	Median	10.52	16.85	7.81	10.00	9.31	14.85	12.30	9.05
	75% Percentile	15.03	26.25	11.92	13.15	10.88	19.53	14.00	12.05
	Maximum	17.30	42.00	22.10	16.20	12.00	22.50	22.60	14.40
Values are reported as percentage of all single cells.									

Supplementary Table 9 – Descriptive statistics: frequency of intermediate monocytes at longitudinal time points following vaccination

Cohort	Statistical parameter	V1D0	V1D1	V1D3	V1D7	V2D0	V2D1	V2D3	V2D7
MVA-S	Sample size	10	10	10	10	10	10	0	10
	Minimum	0.20	0.37	0.13	0.16	0.07	0.26		0.14
	25% Percentile	0.42	0.58	0.38	0.40	0.38	0.39		0.36
	Median	0.66	0.71	0.55	0.65	0.53	0.75		0.47
	75% Percentile	0.91	0.84	1.29	0.85	0.75	1.47		0.97
	Maximum	1.32	1.70	2.61	1.19	1.65	2.23		1.73
MVA-ST	Sample size	14	14	15	15	14	15	0	15
	Minimum	0.13	0.17	0.21	0.15	0.20	0.13		0.17
	25% Percentile	0.26	0.20	0.28	0.31	0.27	0.25		0.29
	Median	0.37	0.39	0.48	0.50	0.47	0.69		0.46
	75% Percentile	0.65	0.80	0.84	0.92	0.92	0.79		0.63
	Maximum	1.04	1.24	2.64	1.54	1.44	1.33		0.90
mRNA	Sample size	9	9	9	9	9	9	8	9
	Minimum	0.21	0.31	0.44	0.19	0.18	0.23	0.74	0.04
	25% Percentile	0.26	0.36	0.56	0.28	0.20	0.27	0.98	0.14
	Median	0.33	0.41	0.90	0.33	0.26	0.44	1.62	0.20
	75% Percentile	0.45	0.56	1.31	0.51	0.48	0.80	2.04	0.45
	Maximum	1.43	1.58	1.56	0.58	0.71	1.11	2.42	0.72
ChAd/ mRNA	Sample size	8	8	8	8	6	8	7	8
	Minimum	0.36	1.08	1.17	0.22	0.28	0.41	1.24	0.28
	25% Percentile	0.45	1.45	1.39	0.32	0.32	0.43	1.38	0.33
	Median	0.59	1.78	1.72	0.42	0.45	0.64	1.95	0.37
	75% Percentile	0.92	3.96	2.32	0.54	0.60	1.22	2.97	0.46
	Maximum	1.77	4.62	3.94	0.88	0.71	1.44	3.75	0.71
Values are reported as percentage of all single cells.									

Supplementary Table 10 – Descriptive statistics: frequency of non-classical monocytes at longitudinal time points following vaccination

Cohort	Statistical parameter	V1D0	V1D1	V1D3	V1D7	V2D0	V2D1	V2D3	V2D7
MVA-S	Sample size	10	10	10	10	10	10	0	10
	Minimum	0.22	0.24	0.23	0.14	0.16	0.25		0.12
	25% Percentile	0.37	0.48	0.36	0.43	0.33	0.49		0.48
	Median	0.54	0.63	0.68	0.66	0.53	0.62		0.65
	75% Percentile	0.85	0.84	0.94	0.78	0.70	0.76		0.77
	Maximum	1.47	1.00	1.14	0.86	1.07	0.90		0.84
MVA-ST	Sample size	14	14	15	15	14	15	0	15
	Minimum	0.09	0.11	0.15	0.12	0.11	0.06		0.10
	25% Percentile	0.19	0.15	0.17	0.18	0.18	0.13		0.16
	Median	0.27	0.23	0.29	0.29	0.29	0.35		0.22
	75% Percentile	0.40	0.39	0.46	0.48	0.42	0.47		0.58
	Maximum	0.86	0.88	0.83	0.93	0.67	0.62		1.07
mRNA	Sample size	9	9	9	9	9	9	8	9
	Minimum	0.19	0.24	0.30	0.26	0.14	0.20	0.35	0.09
	25% Percentile	0.27	0.33	0.36	0.32	0.24	0.32	0.38	0.30
	Median	0.41	0.37	0.46	0.35	0.37	0.52	0.53	0.41
	75% Percentile	0.51	0.42	0.63	0.52	0.43	0.57	0.74	0.79
	Maximum	0.83	0.75	0.83	0.60	0.68	0.80	0.75	1.14
ChAd/ mRNA	Sample size	8	8	8	8	6	8	7	8
	Minimum	0.15	0.25	0.05	0.11	0.10	0.13	0.20	0.15
	25% Percentile	0.30	0.35	0.25	0.14	0.17	0.27	0.35	0.20
	Median	0.48	0.47	0.36	0.24	0.41	0.37	0.48	0.34
	75% Percentile	0.60	0.60	0.58	0.37	0.47	0.55	0.64	0.49
	Maximum	0.63	0.81	0.94	0.45	0.53	0.72	0.85	0.53
Values are reported as percentage of all single cells.									

Supplementary Table 11 – Descriptive statistics: CD40 expression by classical monocytes at longitudinal time points following vaccination

Cohort	Statistical parameter	V1D0	V1D1	V1D3	V1D7	V2D0	V2D1	V2D3	V2D7
MVA-S	Sample size	10	10	10	10	10	10	0	10
	Minimum	1.00	0.55	0.51	0.67	0.71	0.68		0.60
	25% Percentile	1.00	0.75	0.72	0.74	0.81	0.95		0.87
	Median	1.00	0.91	0.85	0.99	1.04	0.99		1.00
	75% Percentile	1.00	0.98	1.09	1.07	1.15	1.17		1.11
	Maximum	1.00	1.21	1.24	1.11	1.47	1.36		1.60
MVA-ST	Sample size	14	14	14	14	13	14	0	14
	Minimum	1.00	0.85	0.65	0.85	0.61	0.80		0.78
	25% Percentile	1.00	0.88	0.89	0.89	0.92	0.96		0.86
	Median	1.00	1.00	0.99	0.99	1.04	1.05		0.97
	75% Percentile	1.00	1.07	1.25	1.31	1.08	1.44		1.17
	Maximum	1.00	1.64	1.53	1.96	1.42	1.80		1.28
mRNA	Sample size	9	9	9	9	9	9	8	9
	Minimum	1.00	0.83	0.73	0.48	0.58	0.86	1.01	0.44
	25% Percentile	1.00	0.88	0.80	0.79	0.87	1.40	1.15	0.90
	Median	1.00	0.89	0.94	1.08	1.18	2.10	1.75	1.02
	75% Percentile	1.00	1.13	1.18	1.24	1.28	2.38	2.08	1.17
	Maximum	1.00	1.22	1.26	1.43	1.45	2.81	2.15	1.67
ChAd/ mRNA	Sample size	8	8	8	8	6	8	7	8
	Minimum	1.00	1.88	1.31	0.81	0.89	1.30	1.93	0.79
	25% Percentile	1.00	2.02	1.45	1.00	1.06	1.33	2.16	1.03
	Median	1.00	2.47	1.65	1.17	1.14	1.74	2.31	1.22
	75% Percentile	1.00	2.82	2.21	1.27	1.23	2.23	3.07	1.33
	Maximum	1.00	3.45	3.19	1.35	1.26	2.94	4.57	1.36
Values are reported as fold change compared to baseline (V1D0) of the median fluorescence intensity (MFI).									

Supplementary Table 12 – Descriptive statistics: CD40 expression by intermediate monocytes at longitudinal time points following vaccination

Cohort	Statistical parameter	V1D0	V1D1	V1D3	V1D7	V2D0	V2D1	V2D3	V2D7
MVA-S	Sample size	10	10	10	10	10	10	0	10
	Minimum	1.00	0.67	0.94	0.74	0.76	0.89		0.83
	25% Percentile	1.00	0.91	0.95	0.84	0.98	0.98		0.91
	Median	1.00	0.98	1.09	0.94	1.02	1.03		1.09
	75% Percentile	1.00	1.02	1.16	1.08	1.19	1.17		1.15
	Maximum	1.00	1.10	1.26	1.23	1.35	1.34		1.28
MVA-ST	Sample size	14	14	14	14	13	14	0	14
	Minimum	1.00	0.85	0.71	0.89	0.83	0.76		0.80
	25% Percentile	1.00	0.89	0.95	0.95	0.93	0.98		0.90
	Median	1.00	0.96	1.06	1.03	0.96	1.01		0.96
	75% Percentile	1.00	1.07	1.16	1.25	1.02	1.11		1.03
	Maximum	1.00	1.39	1.70	1.83	1.11	1.60		1.28
mRNA	Sample size	9	9	9	9	9	9	8	9
	Minimum	1.00	0.75	0.90	0.55	0.59	0.80	1.07	0.54
	25% Percentile	1.00	0.89	0.93	0.87	0.91	1.39	1.39	0.92
	Median	1.00	0.98	1.01	0.94	1.05	1.47	1.82	1.13
	75% Percentile	1.00	1.07	1.32	1.16	1.25	1.79	2.93	1.28
	Maximum	1.00	1.32	1.50	1.50	1.35	2.52	3.03	1.40
ChAd/ mRNA	Sample size	8	8	8	8	6	8	7	8
	Minimum	1.00	1.32	1.38	0.75	0.96	1.16	1.96	0.85
	25% Percentile	1.00	1.48	1.82	0.96	1.01	1.25	2.07	1.06
	Median	1.00	1.81	2.02	1.09	1.12	1.55	2.66	1.14
	75% Percentile	1.00	1.92	2.28	1.22	1.18	1.74	2.88	1.26
	Maximum	1.00	2.09	4.16	1.34	1.26	1.92	4.95	1.36
Values are reported as fold change compared to baseline (V1D0) of the median fluorescence intensity (MFI).									

Supplementary Table 13 – Descriptive statistics: CD40 expression by non-classical monocytes at longitudinal time points following vaccination

Cohort	Statistical parameter	V1D0	V1D1	V1D3	V1D7	V2D0	V2D1	V2D3	V2D7
MVA-S	Sample size	10	10	10	10	10	10	0	10
	Minimum	1.00	0.68	0.73	0.75	0.80	0.76		0.68
	25% Percentile	1.00	0.77	0.89	0.82	0.97	0.83		0.90
	Median	1.00	0.90	1.05	0.89	1.07	0.94		0.95
	75% Percentile	1.00	1.01	1.11	1.00	1.26	1.06		1.09
	Maximum	1.00	1.17	1.27	1.07	1.55	1.19		1.46
MVA-ST	Sample size	14	14	14	14	13	14	0	14
	Minimum	1.00	0.81	0.85	0.80	0.79	0.76		0.80
	25% Percentile	1.00	0.90	0.94	0.97	0.82	0.85		0.89
	Median	1.00	0.99	1.01	1.02	0.95	1.04		0.96
	75% Percentile	1.00	1.11	1.28	1.26	1.05	1.16		1.02
	Maximum	1.00	1.23	1.61	1.50	1.11	1.56		1.68
mRNA	Sample size	9	9	9	9	9	9	8	9
	Minimum	1.00	0.74	0.78	0.75	0.84	0.68	1.02	0.75
	25% Percentile	1.00	0.80	0.88	0.93	0.98	1.11	1.14	0.92
	Median	1.00	0.88	0.98	1.01	1.05	1.24	1.51	1.19
	75% Percentile	1.00	1.02	1.25	1.34	1.44	1.62	1.95	1.44
	Maximum	1.00	1.06	1.68	1.71	1.77	1.72	2.43	1.76
ChAd/ mRNA	Sample size	8	8	8	8	6	8	7	8
	Minimum	1.00	1.22	0.64	0.46	1.08	1.06	1.88	1.13
	25% Percentile	1.00	1.32	1.52	0.93	1.09	1.15	1.90	1.19
	Median	1.00	1.68	2.00	1.06	1.10	1.42	2.27	1.27
	75% Percentile	1.00	2.19	2.05	1.18	2.44	1.70	2.67	1.41
	Maximum	1.00	8.56	3.06	1.51	5.84	5.11	8.17	3.72
Values are reported as fold change compared to baseline (V1D0) of the median fluorescence intensity (MFI).									

Supplementary Table 14 – Descriptive statistics: frequency of CD16+ dendritic cells at longitudinal time points following vaccination

Cohort	Statistical parameter	V1D0	V1D1	V1D3	V1D7	V2D0	V2D1	V2D3	V2D7
MVA-S	Sample size	10	10	10	10	10	10	0	10
	Minimum	0.20	0.18	0.27	0.11	0.22	0.21		0.09
	25% Percentile	0.36	0.60	0.45	0.55	0.31	0.41		0.40
	Median	0.73	0.86	0.65	0.79	0.50	0.86		0.86
	75% Percentile	1.42	1.29	0.92	1.36	1.37	1.23		1.35
	Maximum	1.62	1.77	1.33	1.75	1.93	1.65		1.68
MVA-ST	Sample size	14	14	15	15	14	15	0	15
	Minimum	0.06	0.06	0.08	0.07	0.06	0.05		0.05
	25% Percentile	0.25	0.17	0.29	0.28	0.29	0.22		0.30
	Median	0.56	0.54	0.63	0.60	0.55	0.57		0.43
	75% Percentile	0.83	0.87	0.87	1.09	1.01	0.99		0.88
	Maximum	1.61	1.64	1.25	1.81	1.20	1.16		1.38
mRNA	Sample size	9	9	9	9	9	9	8	9
	Minimum	0.10	0.06	0.10	0.05	0.08	0.09	0.15	0.04
	25% Percentile	0.21	0.23	0.20	0.19	0.21	0.29	0.25	0.26
	Median	0.33	0.41	0.48	0.64	0.70	0.73	0.58	0.41
	75% Percentile	0.75	0.77	0.85	0.77	0.88	1.23	1.07	1.02
	Maximum	1.66	0.94	1.13	1.16	1.67	1.48	1.38	1.39
ChAd/ mRNA	Sample size	8	8	8	8	6	8	7	8
	Minimum	0.28	0.31	0.15	0.06	0.20	0.27	0.31	0.22
	25% Percentile	0.34	0.44	0.28	0.07	0.30	0.32	0.42	0.24
	Median	0.66	0.85	0.42	0.15	0.42	0.63	0.75	0.35
	75% Percentile	0.95	1.61	0.83	0.43	0.70	0.89	0.81	0.75
	Maximum	1.51	1.98	0.92	0.62	0.73	1.43	1.54	0.87
Values are reported as percentage of all single cells.									

Supplementary Table 15 – Descriptive statistics: frequency of CD141+ dendritic cells at longitudinal time points following vaccination

Cohort	Statistical parameter	V1D0	V1D1	V1D3	V1D7	V2D0	V2D1	V2D3	V2D7
MVA-S	Sample size	10	10	10	10	10	10	0	10
	Minimum	0.05	0.05	0.04	0.04	0.05	0.03		0.04
	25% Percentile	0.07	0.05	0.06	0.05	0.06	0.05		0.05
	Median	0.08	0.07	0.07	0.08	0.08	0.08		0.07
	75% Percentile	0.12	0.10	0.09	0.10	0.10	0.10		0.09
	Maximum	0.15	0.10	0.11	0.14	0.15	0.11		0.13
MVA-ST	Sample size	14	14	15	15	14	15	0	15
	Minimum	0.04	0.02	0.03	0.03	0.04	0.01		0.04
	25% Percentile	0.05	0.03	0.03	0.04	0.05	0.03		0.04
	Median	0.05	0.04	0.05	0.05	0.06	0.04		0.06
	75% Percentile	0.07	0.05	0.06	0.07	0.07	0.05		0.06
	Maximum	0.11	0.08	0.11	0.09	0.12	0.11		0.11
mRNA	Sample size	9	9	9	9	9	9	8	9
	Minimum	0.03	0.02	0.02	0.02	0.03	0.02	0.03	0.02
	25% Percentile	0.04	0.03	0.03	0.03	0.03	0.02	0.04	0.03
	Median	0.05	0.04	0.04	0.04	0.05	0.03	0.05	0.04
	75% Percentile	0.07	0.05	0.05	0.05	0.05	0.04	0.06	0.05
	Maximum	0.07	0.08	0.06	0.07	0.06	0.05	0.08	0.07
ChAd/ mRNA	Sample size	8	8	8	8	6	8	7	8
	Minimum	0.03	0.03	0.03	0.03	0.04	0.02	0.03	0.03
	25% Percentile	0.05	0.04	0.03	0.04	0.04	0.03	0.04	0.03
	Median	0.06	0.07	0.04	0.05	0.07	0.04	0.05	0.04
	75% Percentile	0.07	0.08	0.06	0.08	0.08	0.06	0.08	0.07
	Maximum	0.09	0.17	0.06	0.09	0.09	0.06	0.11	0.09
Values are reported as percentage of all single cells.									

Supplementary Table 16 – Descriptive statistics: frequency of CD1c+ dendritic cells at longitudinal time points following vaccination

**Cohort**	**Statistical parameter**	**V1D0**	**V1D1**	**V1D3**	**V1D7**	**V2D0**	**V2D1**	**V2D3**	**V2D7**
MVA-S	Sample size	10	10	10	10	10	10	0	10
	Minimum	0.13	0.24	0.13	0.19	0.19	0.18		0.11
	25% Percentile	0.25	0.26	0.24	0.25	0.28	0.32		0.27
	Median	0.33	0.35	0.34	0.31	0.34	0.36		0.34
	75% Percentile	0.44	0.41	0.43	0.37	0.37	0.40		0.43
	Maximum	0.48	0.49	0.52	0.47	0.45	0.44		0.52
MVA-ST	Sample size	14	14	15	15	14	15	0	15
	Minimum	0.25	0.17	0.17	0.22	0.25	0.14		0.22
	25% Percentile	0.31	0.24	0.25	0.25	0.30	0.23		0.31
	Median	0.39	0.30	0.29	0.30	0.38	0.31		0.34
	75% Percentile	0.45	0.34	0.39	0.35	0.51	0.38		0.42
	Maximum	0.55	0.42	0.66	0.41	0.55	0.54		0.71
mRNA	Sample size	9	9	9	9	9	9	8	9
	Minimum	0.13	0.20	0.15	0.13	0.18	0.13	0.14	0.10
	25% Percentile	0.21	0.21	0.21	0.20	0.21	0.16	0.26	0.19
	Median	0.29	0.26	0.32	0.26	0.33	0.28	0.31	0.30
	75% Percentile	0.31	0.34	0.38	0.33	0.39	0.33	0.49	0.39
	Maximum	0.60	0.49	0.50	0.49	0.49	0.57	0.76	0.47
ChAd/ mRNA	Sample size	8	8	8	8	6	8	7	8
	Minimum	0.21	0.15	0.24	0.23	0.21	0.19	0.31	0.29
	25% Percentile	0.30	0.16	0.31	0.25	0.38	0.25	0.32	0.32
	Median	0.38	0.24	0.36	0.35	0.51	0.31	0.45	0.35
	75% Percentile	0.41	0.25	0.42	0.45	0.58	0.38	0.57	0.47
	Maximum	0.70	0.27	0.45	0.51	0.62	0.46	0.81	0.65
Values are reported as percentage of all single cells.									

Supplementary Table 17 – Descriptive statistics: frequency of plasmacytoid dendritic cells (pDCs) at longitudinal time points following vaccination

Cohort	Statistical parameter	V1D0	V1D1	V1D3	V1D7	V2D0	V2D1	V2D3	V2D7
MVA-S	Sample size	10	10	10	10	10	10	0	10
	Minimum	0.28	0.22	0.27	0.24	0.23	0.19		0.15
	25% Percentile	0.34	0.38	0.31	0.27	0.33	0.27		0.21
	Median	0.37	0.42	0.43	0.39	0.42	0.37		0.47
	75% Percentile	0.60	0.52	0.57	0.47	0.51	0.48		0.53
	Maximum	0.64	0.77	1.00	0.92	0.72	0.93		0.61
MVA-ST	Sample size	14	14	15	15	14	15	0	15
	Minimum	0.15	0.12	0.19	0.16	0.20	0.18		0.17
	25% Percentile	0.29	0.26	0.33	0.35	0.28	0.30		0.33
	Median	0.39	0.34	0.41	0.40	0.38	0.35		0.40
	75% Percentile	0.46	0.53	0.47	0.47	0.50	0.53		0.43
	Maximum	0.76	0.56	0.62	0.54	0.53	0.73		0.65
mRNA	Sample size	9	9	9	9	9	9	8	9
	Minimum	0.17	0.13	0.13	0.11	0.18	0.19	0.24	0.19
	25% Percentile	0.24	0.22	0.34	0.18	0.21	0.28	0.29	0.20
	Median	0.29	0.48	0.49	0.29	0.27	0.38	0.39	0.30
	75% Percentile	0.33	0.51	0.59	0.38	0.32	0.46	0.46	0.39
	Maximum	0.40	0.77	0.78	0.60	0.34	0.68	0.75	0.42
ChAd/ mRNA	Sample size	8	8	8	8	6	8	7	8
	Minimum	0.32	0.41	0.29	0.25	0.29	0.32	0.28	0.18
	25% Percentile	0.39	0.53	0.34	0.26	0.31	0.39	0.35	0.23
	Median	0.48	0.74	0.41	0.33	0.41	0.42	0.41	0.37
	75% Percentile	0.70	0.94	0.50	0.38	0.50	0.56	0.43	0.39
	Maximum	0.87	1.30	0.58	0.39	0.69	0.84	0.55	0.40
Values are reported as percentage of all single cells.									

Supplementary Table 18 – Descriptive statistics: CD40 expression of CD16+ dendritic cells at longitudinal time points following vaccination

Cohort	Statistical parameter	V1D0	V1D1	V1D3	V1D7	V2D0	V2D1	V2D3	V2D7
MVA-S	Sample size	10	10	10	10	10	10	0	10
	Minimum	1.00	0.63	0.56	0.70	0.65	0.54		0.71
	25% Percentile	1.00	0.71	0.88	0.71	0.79	0.78		0.78
	Median	1.00	0.80	1.05	0.87	1.04	0.95		1.00
	75% Percentile	1.00	1.00	1.13	1.02	1.38	1.05		1.17
	Maximum	1.00	1.10	1.77	1.09	1.50	1.54		1.61
MVA-ST	Sample size	14	14	14	14	13	14	0	14
	Minimum	1.00	0.73	0.72	0.87	0.68	0.80		0.89
	25% Percentile	1.00	0.89	0.84	0.91	0.87	0.84		0.97
	Median	1.00	1.00	0.99	1.02	1.01	1.04		1.07
	75% Percentile	1.00	1.08	1.26	1.26	1.14	1.31		1.29
	Maximum	1.00	1.29	1.55	1.56	1.20	1.61		1.61
mRNA	Sample size	9	9	9	9	9	9	8	9
	Minimum	1.00	0.60	0.79	0.80	0.63	0.73	0.97	0.78
	25% Percentile	1.00	0.86	0.88	1.12	1.05	1.44	1.29	1.09
	Median	1.00	0.98	1.18	1.28	1.21	1.71	2.29	1.51
	75% Percentile	1.00	1.11	1.37	1.38	1.44	1.99	3.03	1.92
	Maximum	1.00	1.45	1.71	1.63	2.36	2.69	3.26	2.30
ChAd/ mRNA	Sample size	8	8	8	8	6	8	7	8
	Minimum	1.00	1.31	1.49	1.02	1.08	1.04	1.91	1.17
	25% Percentile	1.00	1.50	1.76	1.26	1.09	1.14	1.99	1.36
	Median	1.00	1.75	2.17	1.39	1.24	1.49	2.38	1.41
	75% Percentile	1.00	2.78	3.27	1.63	1.28	1.82	2.55	1.66
	Maximum	1.00	2.81	5.10	1.71	1.29	2.14	4.89	1.99
Values are reported as fold change compared to baseline (V1D0) of the median fluorescence intensity (MFI).									

Supplementary Table 19 – Descriptive statistics: CD40 expression of CD141+ dendritic cells at longitudinal time points following vaccination

Cohort	Statistical parameter	V1D0	V1D1	V1D3	V1D7	V2D0	V2D1	V2D3	V2D7
MVA-S	Sample size	10	10	10	10	10	10	0	10
	Minimum	1.00	0.49	0.22	0.47	0.78	0.59		0.75
	25% Percentile	1.00	0.58	0.77	0.65	0.84	0.69		0.81
	Median	1.00	0.70	0.88	0.82	0.94	0.79		0.90
	75% Percentile	1.00	0.96	1.09	0.99	1.29	1.06		1.11
	Maximum	1.00	1.32	1.45	1.27	1.45	1.40		1.50
MVA-ST	Sample size	14	14	14	14	13	14	0	14
	Minimum	1.00	0.64	0.51	0.64	0.68	0.54		0.49
	25% Percentile	1.00	0.78	0.86	0.83	0.85	0.70		0.85
	Median	1.00	1.01	1.02	1.01	0.95	0.99		0.96
	75% Percentile	1.00	1.20	1.18	1.17	1.20	1.18		1.42
	Maximum	1.00	1.39	1.39	1.48	1.25	1.39		2.41
mRNA	Sample size	9	9	9	9	9	9	8	9
	Minimum	1.00	0.33	0.38	0.51	0.55	0.38	0.36	0.67
	25% Percentile	1.00	0.60	0.52	0.86	0.78	0.51	0.50	0.77
	Median	1.00	0.68	0.62	0.89	0.84	0.71	0.76	0.83
	75% Percentile	1.00	0.78	0.82	1.13	1.10	0.90	0.97	1.03
	Maximum	1.00	0.85	0.95	1.18	1.24	1.24	0.99	1.67
ChAd/ mRNA	Sample size	8	8	8	8	6	8	7	8
	Minimum	1.00	0.99	0.71	0.87	0.95	0.54	0.85	0.82
	25% Percentile	1.00	1.10	0.90	0.99	1.02	0.87	0.93	0.89
	Median	1.00	1.66	1.00	1.02	1.07	1.07	0.99	1.11
	75% Percentile	1.00	2.55	1.12	1.18	1.28	1.10	1.18	1.29
	Maximum	1.00	3.44	1.23	1.63	1.53	1.39	1.40	1.37
Values are reported as fold change compared to baseline (V1D0) of the median fluorescence intensity (MFI).									

Supplementary Table 20 – Descriptive statistics: CD40 expression of CD1c+ dendritic cells at longitudinal time points following vaccination

Cohort	Statistical parameter	V1D0	V1D1	V1D3	V1D7	V2D0	V2D1	V2D3	V2D7
MVA-S	Sample size	10	10	10	10	10	10	0	10
	Minimum	1.00	0.34	0.24	0.38	0.57	0.36		0.42
	25% Percentile	1.00	0.52	0.59	0.58	0.74	0.63		0.55
	Median	1.00	0.60	0.76	0.77	0.99	0.76		0.87
	75% Percentile	1.00	0.92	0.94	0.87	1.16	0.81		0.93
	Maximum	1.00	1.09	1.14	0.90	1.27	1.08		1.74
MVA-ST	Sample size	14	14	14	14	13	14	0	14
	Minimum	1.00	0.44	0.41	0.38	0.41	0.56		0.60
	25% Percentile	1.00	0.89	0.81	0.83	0.90	0.87		0.89
	Median	1.00	1.04	0.95	0.92	1.08	1.12		0.97
	75% Percentile	1.00	1.20	1.11	1.12	1.17	1.44		1.22
	Maximum	1.00	1.58	1.88	1.47	1.42	2.22		1.76
mRNA	Sample size	9	9	9	9	9	9	8	9
	Minimum	1.00	0.43	0.52	0.68	0.73	0.79	0.68	0.44
	25% Percentile	1.00	0.61	0.58	0.82	0.98	1.02	0.73	0.78
	Median	1.00	0.79	0.66	1.32	1.32	1.38	0.93	0.89
	75% Percentile	1.00	0.91	0.76	1.39	1.65	1.89	1.19	1.19
	Maximum	1.00	1.15	1.50	1.44	2.13	3.00	1.43	3.24
ChAd/ mRNA	Sample size	8	8	8	8	6	8	7	8
	Minimum	1.00	1.93	0.81	0.86	0.91	0.86	1.36	0.78
	25% Percentile	1.00	2.03	1.12	0.97	0.93	1.05	1.46	0.91
	Median	1.00	2.31	1.51	1.23	1.14	1.40	1.71	1.26
	75% Percentile	1.00	4.82	1.85	1.45	1.22	1.87	1.86	1.60
	Maximum	1.00	7.26	2.66	1.61	1.23	2.60	2.15	2.11
Values are reported as fold change compared to baseline (V1D0) of the median fluorescence intensity (MFI).									

Supplementary Table 21 – Descriptive statistics: CD40 expression of plasmacytoid dendritic cells (pDCs) at longitudinal time points following vaccination

Cohort	Statistical parameter	V1D0	V1D1	V1D3	V1D7	V2D0	V2D1	V2D3	V2D7
MVA-S	Sample size	10	10	10	10	10	10	0	10
	Minimum	1.00	0.47	0.42	0.49	0.59	0.42		0.60
	25% Percentile	1.00	0.67	0.62	0.58	0.94	0.75		0.71
	Median	1.00	0.80	0.87	0.83	1.05	0.85		0.88
	75% Percentile	1.00	0.89	1.01	1.05	1.25	1.06		1.24
	Maximum	1.00	1.17	1.24	1.12	1.94	1.12		1.84
MVA-ST	Sample size	14	14	14	14	13	14	0	14
	Minimum	1.00	0.70	0.76	0.70	0.77	0.79		0.74
	25% Percentile	1.00	0.84	0.84	0.85	0.86	0.92		0.91
	Median	1.00	1.01	0.96	0.99	0.97	0.99		1.01
	75% Percentile	1.00	1.08	1.04	1.10	0.99	1.05		1.06
	Maximum	1.00	1.16	1.11	1.24	1.26	1.24		1.17
mRNA	Sample size	9	9	9	9	9	9	8	9
	Minimum	1.00	0.32	0.37	0.57	0.82	0.44	0.44	0.62
	25% Percentile	1.00	0.55	0.50	0.84	0.94	0.48	0.72	0.78
	Median	1.00	0.62	0.59	1.14	1.18	0.76	0.80	0.91
	75% Percentile	1.00	0.74	0.94	1.59	1.47	0.95	0.90	1.33
	Maximum	1.00	0.94	1.73	2.13	2.02	1.42	1.04	2.12
ChAd/ mRNA	Sample size	8	8	8	8	6	8	7	8
	Minimum	1.00	0.65	0.87	1.00	0.90	0.90	0.88	0.82
	25% Percentile	1.00	0.68	0.98	1.08	0.95	0.91	0.94	0.86
	Median	1.00	1.14	1.20	1.14	0.99	1.04	0.97	1.00
	75% Percentile	1.00	1.19	1.46	1.34	1.07	1.34	1.32	1.16
	Maximum	1.00	1.41	1.70	1.44	1.26	1.68	1.43	1.50

Values are reported as fold change compared to baseline (V1D0) of the median fluorescence intensity (MFI).

Supplementary Table 22 – Descriptive statistics: frequency of cTFH17 cells at longitudinal time points following vaccination

Cohort	Statistical parameter	V1D0	V1D7	V1D14	V2D0	V2D7	V2D14
MVA-S	Sample size	10	10	10	10	10	10
	Minimum	10.20	14.40	9.63	11.50	6.38	11.30
	25% Percentile	14.63	16.30	13.73	15.78	14.63	14.58
	Median	17.70	18.45	16.95	17.05	16.65	16.40
	75% Percentile	21.43	21.08	19.15	19.45	21.05	20.15
	Maximum	26.20	25.90	26.50	24.00	23.00	25.70
MVA-ST	Sample size	14	15	15	14	15	11
	Minimum	9.92	11.20	10.70	9.42	8.94	7.80
	25% Percentile	14.43	15.50	14.60	13.53	14.80	12.10
	Median	19.10	17.90	18.50	18.90	20.20	17.45
	75% Percentile	25.13	25.50	25.30	23.00	23.40	23.40
	Maximum	32.00	32.60	30.40	31.20	29.90	27.10
mRNA	Sample size	9	9	0	9	9	0
	Minimum	12.30	13.60		10.20	13.10	
	25% Percentile	14.40	15.20		12.90	15.20	
	Median	20.60	20.40		16.30	17.20	
	75% Percentile	26.20	26.20		25.10	24.85	
	Maximum	44.10	54.10		44.70	34.30	
ChAd/ mRNA	Sample size	8	8	7	6	8	8
	Minimum	17.60	13.40	15.10	15.60	13.80	14.80
	25% Percentile	17.78	15.65	17.30	16.50	15.38	15.78
	Median	19.65	16.35	18.90	18.00	16.30	17.60
	75% Percentile	21.75	19.43	19.90	22.38	23.35	22.53
	Maximum	32.70	20.60	23.30	27.70	25.70	25.00
Values are reported as percentage of the cTFH cell population. cTFH cell = circulating T follicular helper cell.							

Supplementary Table 23 – Descriptive statistics: frequency of ICOS+ cTFH17 cells at longitudinal time points following vaccination

Cohort	Statistical parameter	V1D0	V1D7	V1D14	V2D0	V2D7	V2D14
MVA-S	Sample size	10	10	10	10	10	10
	Minimum	1.76	3.56	2.52	2.77	4.23	2.31
	25% Percentile	4.49	4.91	3.14	4.51	5.14	4.34
	Median	6.25	7.32	5.10	6.84	6.74	5.08
	75% Percentile	8.62	8.22	8.92	9.06	8.12	8.61
	Maximum	10.80	14.60	9.42	11.90	9.56	10.10
MVA-ST	Sample size	14	15	15	14	15	11
	Minimum	5.73	2.10	8.88	5.15	5.55	4.91
	25% Percentile	10.33	14.00	10.90	12.18	12.60	14.70
	Median	15.80	17.80	14.90	16.00	19.20	20.80
	75% Percentile	18.70	21.70	24.30	23.88	25.00	23.50
	Maximum	36.50	30.90	33.60	29.60	30.50	24.80
mRNA	Sample size	9	9	0	9	9	0
	Minimum	9.73	5.79		5.07	10.50	
	25% Percentile	9.87	7.87		8.73	12.55	
	Median	10.90	13.60		11.00	17.00	
	75% Percentile	16.75	24.55		16.35	21.30	
	Maximum	26.80	33.20		29.40	28.40	
ChAd/ mRNA	Sample size	8	8	7	6	8	8
	Minimum	5.46	6.85	11.10	12.20	5.09	5.30
	25% Percentile	10.06	12.33	14.40	15.58	11.38	12.40
	Median	16.40	17.70	16.10	18.35	16.00	16.15
	75% Percentile	21.75	22.45	16.90	23.35	23.35	21.08
	Maximum	30.90	29.80	24.50	25.60	32.60	27.70
Values are reported as fold change compared to baseline (V1D0) of the frequency of ICOS+ cells within cTFH17 cells.							

Supplementary Table 24 – Descriptive statistics: frequency of CD38+ cTFH17 cells at longitudinal time points following vaccination

Cohort	Statistical parameter	V1D0	V1D7	V1D14	V2D0	V2D7	V2D14
MVA-S	Sample size	10	10	10	10	10	10
	Minimum	9.18	8.70	10.30	7.22	9.77	10.40
	25% Percentile	11.90	11.68	12.83	8.97	11.19	10.58
	Median	16.75	14.90	17.30	15.10	14.70	16.40
	75% Percentile	20.90	20.58	21.83	18.75	17.15	22.65
	Maximum	33.00	30.50	33.20	21.80	27.60	27.60
MVA-ST	Sample size	14	15	15	14	15	11
	Minimum	1.32	1.89	1.61	3.07	3.01	1.84
	25% Percentile	3.00	5.14	4.87	4.53	4.29	2.85
	Median	4.66	6.23	5.99	5.75	6.08	4.31
	75% Percentile	6.89	7.57	12.00	8.12	9.25	8.87
	Maximum	18.90	16.00	14.50	15.40	10.70	11.20
mRNA	Sample size	9	9	0	9	9	0
	Minimum	3.66	3.17		5.80	7.09	
	25% Percentile	5.27	8.32		6.64	8.09	
	Median	9.40	11.30		7.45	9.49	
	75% Percentile	12.75	14.40		14.35	16.00	
	Maximum	14.80	15.90		26.90	18.80	
ChAd/ mRNA	Sample size	8	8	7	6	8	8
	Minimum	4.84	4.86	4.37	3.34	5.60	4.52
	25% Percentile	5.34	7.22	4.42	6.33	6.28	5.86
	Median	6.99	8.98	7.80	8.30	7.54	6.96
	75% Percentile	11.15	11.66	9.27	11.98	9.43	8.90
	Maximum	13.10	13.90	11.00	13.40	11.10	9.98
Values are reported as fold change compared to baseline (V1D0) of the frequency of CD38+ cells within cTFH17 cells.							

Supplementary Table 25 – Descriptive statistics: frequency of cTFH1 cells at longitudinal time points following vaccination

Cohort	Statistical parameter	V1D0	V1D7	V1D14	V2D0	V2D7	V2D14
MVA-S	Sample size	10	10	10	10	10	10
	Minimum	37.80	39.70	38.20	35.60	30.80	34.80
	25% Percentile	40.38	42.05	45.00	45.13	43.90	46.75
	Median	47.15	45.90	49.50	48.45	49.55	48.85
	75% Percentile	51.93	48.35	50.63	51.75	52.28	53.05
	Maximum	55.20	51.30	54.60	53.40	56.70	55.30
MVA-ST	Sample size	14	12	15	14	15	11
	Minimum	29.10	28.70	30.40	31.80	30.80	33.10
	25% Percentile	35.10	34.60	39.10	37.18	39.70	35.10
	Median	38.20	45.60	43.80	41.30	44.60	45.40
	75% Percentile	46.10	53.70	51.30	49.98	54.20	55.20
	Maximum	59.90	57.50	56.60	56.20	57.80	59.20
mRNA	Sample size	9	9	0	9	9	0
	Minimum	11.40	8.26		9.75	17.60	
	25% Percentile	36.20	31.60		38.15	39.00	
	Median	40.30	40.50		43.80	43.80	
	75% Percentile	49.00	48.35		54.20	50.00	
	Maximum	62.20	63.20		63.80	62.20	
ChAd/ mRNA	Sample size	8	8	7	6	8	8
	Minimum	30.00	41.00	34.70	36.20	40.00	41.50
	25% Percentile	36.58	44.58	43.60	37.10	42.10	43.05
	Median	44.90	52.30	44.70	46.50	47.55	46.75
	75% Percentile	47.40	56.50	48.80	48.20	51.33	51.20
	Maximum	49.20	60.00	51.70	49.40	56.90	51.80
Values are reported as percentage of the cTFH cell population. cTFH cell = circulating T follicular helper cell.							

Supplementary Table 26 – Descriptive statistics: frequency of ICOS+ cTFH1 cells at longitudinal time points following vaccination

Cohort	Statistical parameter	V1D0	V1D7	V1D14	V2D0	V2D7	V2D14
MVA-S	Sample size	10	10	10	10	10	10
	Minimum	2.68	2.76	5.47	3.52	5.71	3.76
	25% Percentile	4.33	5.68	5.92	5.34	6.54	4.89
	Median	4.80	7.59	7.64	7.66	7.77	6.69
	75% Percentile	7.33	10.16	9.89	9.63	10.05	8.17
	Maximum	11.30	16.00	16.30	10.30	12.10	9.65
MVA-ST	Sample size	14	15	15	14	15	11
	Minimum	7.13	10.10	10.10	7.91	8.67	6.85
	25% Percentile	9.08	11.20	13.10	11.13	12.00	7.96
	Median	11.90	16.30	16.00	13.75	16.60	13.70
	75% Percentile	15.43	20.20	24.60	15.85	22.80	21.00
	Maximum	29.40	34.70	37.60	22.70	41.80	28.90
mRNA	Sample size	9	9	0	9	9	0
	Minimum	3.53	2.77		5.88	9.90	
	25% Percentile	5.82	9.24		6.91	11.25	
	Median	8.90	12.40		10.80	16.10	
	75% Percentile	14.20	19.30		14.25	22.20	
	Maximum	16.50	24.00		15.20	25.30	
ChAd/ mRNA	Sample size	8	8	7	6	8	8
	Minimum	5.46	13.00	8.58	6.62	4.92	5.51
	25% Percentile	7.83	17.40	8.84	7.33	11.93	9.84
	Median	9.93	32.55	10.90	11.76	16.40	11.25
	75% Percentile	12.58	37.15	15.40	14.10	22.28	16.75
	Maximum	16.20	47.40	17.20	15.00	23.90	17.30
Values are reported as fold change compared to baseline (V1D0) of the frequency of ICOS+ cells within cTFH1 cells.							

Supplementary Table 27 – Descriptive statistics: frequency of CD38+ cTFH1 cells at longitudinal time points following vaccination

Cohort	Statistical parameter	V1D0	V1D7	V1D14	V2D0	V2D7	V2D14
MVA-S	Sample size	10	10	10	10	10	10
	Minimum	13.60	17.20	13.90	9.76	18.70	16.40
	25% Percentile	17.30	18.15	21.18	17.93	19.68	16.98
	Median	19.20	20.85	25.60	22.90	23.40	22.50
	75% Percentile	26.28	31.53	33.45	28.95	31.30	32.80
	Maximum	41.90	42.70	39.20	38.00	47.60	37.80
MVA-ST	Sample size	14	15	15	14	15	11
	Minimum	4.92	5.17	4.84	4.36	8.03	4.21
	25% Percentile	7.16	10.75	7.83	8.32	10.44	6.53
	Median	9.44	13.45	11.25	9.80	14.05	11.60
	75% Percentile	13.23	19.15	20.20	15.05	20.85	13.10
	Maximum	36.10	30.50	35.70	22.50	42.20	24.10
mRNA	Sample size	9	9	0	9	9	0
	Minimum	5.38	7.74		8.46	12.20	
	25% Percentile	8.74	10.05		10.23	14.15	
	Median	11.80	16.60		14.80	17.30	
	75% Percentile	16.00	20.60		18.30	21.10	
	Maximum	19.50	21.50		22.60	23.90	
ChAd/ mRNA	Sample size	8	8	7	6	8	8
	Minimum	3.68	16.80	6.93	3.95	5.16	6.33
	25% Percentile	4.62	20.10	7.11	4.46	9.05	7.79
	Median	8.45	34.45	9.39	8.57	14.15	9.93
	75% Percentile	12.65	41.90	12.40	13.53	18.63	12.53
	Maximum	15.20	46.80	12.40	14.80	21.50	13.90

Values are reported as fold change compared to baseline (V1D0) of the frequency of CD38+ cells within cTFH1 cells.

Supplementary Table 28 – Descriptive statistics: frequency of cTFH2 cells at longitudinal time points following vaccination

Cohort	Statistical parameter	V1D0	V1D7	V1D14	V2D0	V2D7	V2D14
MVA-S	Sample size	10.	10	10	10	10	10
	Minimum	9.82	8.17	8.80	10.50	10.20	12.90
	25% Percentile	14.25	11.95	14.73	12.45	13.53	13.33
	Median	17.35	15.00	18.40	18.10	18.75	18.10
	75% Percentile	23.68	19.38	23.70	23.35	22.70	21.83
	Maximum	27.50	25.50	28.60	29.90	32.10	28.10
MVA-ST	Sample size	14	15	15	14	15	11
	Minimum	9.91	12.80	11.30	10.40	11.30	15.90
	25% Percentile	16.65	13.80	16.40	16.68	13.80	19.00
	Median	19.10	19.60	20.10	20.35	18.00	20.80
	75% Percentile	22.15	21.50	24.10	22.00	21.90	24.00
	Maximum	43.90	23.40	26.80	29.50	28.70	31.70
mRNA	Sample size	9	9	0	9	9	0
	Minimum	11.00	9.91		13.60	14.70	
	25% Percentile	17.95	15.20		15.55	18.75	
	Median	24.70	26.50		22.00	23.20	
	75% Percentile	30.25	31.35		29.15	28.35	
	Maximum	38.00	39.10		30.60	29.30	
ChAd/ mRNA	Sample size	8	8	7	6	8	8
	Minimum	15.30	13.80	18.40	15.50	15.10	16.10
	25% Percentile	17.38	14.30	20.50	17.08	15.68	16.85
	Median	20.05	17.95	21.00	19.00	17.90	18.50
	75% Percentile	25.03	19.33	25.30	23.55	20.58	24.80
	Maximum	26.70	21.90	25.50	25.20	23.30	26.10
Values are reported as percentage of the cTFH cell population. cTFH cell = circulating T follicular helper cell.							

Supplementary Table 29 – Descriptive statistics: frequency of ICOS+ cTFH2 cells at longitudinal time points following vaccination

Cohort	Statistical parameter	V1D0	V1D7	V1D14	V2D0	V2D7	V2D14
MVA-S	Sample size	10	10	10	10	10	10
	Minimum	4.14	5.12	3.87	4.50	4.89	4.96
	25% Percentile	4.62	6.06	4.96	5.97	5.59	5.78
	Median	7.80	7.65	7.48	7.68	8.24	7.25
	75% Percentile	9.82	12.20	8.82	9.54	9.71	7.82
	Maximum	12.90	23.80	12.90	12.00	11.00	11.80
MVA-ST	Sample size	14	15	15	14	15	11
	Minimum	3.08	4.71	4.59	4.60	6.50	5.84
	25% Percentile	9.68	13.30	11.90	11.55	9.38	8.48
	Median	17.05	17.90	19.00	17.15	18.90	16.20
	75% Percentile	26.13	28.80	25.90	25.90	29.50	23.30
	Maximum	32.30	34.40	30.80	37.10	42.10	37.60
mRNA	Sample size	9	9		9	9	
	Minimum	6.09	7.76		8.08	10.40	
	25% Percentile	7.97	9.45		10.47	13.30	
	Median	11.60	12.40		12.20	16.50	
	75% Percentile	17.30	20.45		19.10	20.40	
	Maximum	20.20	26.40		20.30	22.90	
ChAd/ mRNA	Sample size	8	8	7	6	8	8
	Minimum	6.57	5.08	11.00	9.03	5.34	4.80
	25% Percentile	12.18	10.13	13.70	11.33	13.90	11.33
	Median	13.80	15.25	15.80	16.10	16.15	13.70
	75% Percentile	21.08	20.20	20.00	20.50	23.10	20.63
	Maximum	27.10	25.20	25.20	26.80	26.10	26.20
Values are reported as fold change compared to baseline (V1D0) of the frequency of ICOS+ cells within cTFH2 cells.							

Supplementary Table 30 – Descriptive statistics: frequency of CD38+ cTFH2 cells at longitudinal time points following vaccination

Cohort	Statistical parameter	V1D0	V1D7	V1D14	V2D0	V2D7	V2D14
MVA-S	Sample size	10	10	10	10	10	10
	Minimum	11.00	12.10	11.10	9.46	11.80	12.90
	25% Percentile	14.25	15.43	12.70	14.15	14.75	14.98
	Median	20.20	18.80	18.20	17.80	16.85	17.00
	75% Percentile	23.38	23.48	22.90	19.25	19.50	22.90
	Maximum	27.70	25.80	28.50	27.10	28.80	29.60
MVA-ST	Sample size	14	15	15	14	15	11
	Minimum	2.95	2.35	1.62	2.48	2.81	2.97
	25% Percentile	4.66	5.53	4.50	3.97	6.53	3.90
	Median	5.53	7.71	7.84	6.38	7.37	5.99
	75% Percentile	9.46	10.20	11.60	7.39	10.20	9.24
	Maximum	18.30	17.30	15.90	11.20	15.00	10,0
mRNA	Sample size	9	9	0	9	9	0
	Minimum	4.47	6.93		8.09	9.61	
	25% Percentile	7.24	7.44		10.20	11.10	
	Median	11.10	13.50		11.00	13.00	
	75% Percentile	16.90	22.60		19.10	17.45	
	Maximum	25.10	26.90		27.10	22.60	
ChAd/ mRNA	Sample size	8	8	7	6	8	8
	Minimum	6.63	5.98	7.73	4.79	7.63	7.36
	25% Percentile	7.32	7.81	8.35	5.65	8.91	9.00
	Median	9.99	11.90	8.97	10.04	9.41	9.50
	75% Percentile	13.85	13.43	12.40	13.63	12.35	10.88
	Maximum	14.20	14.40	13.00	15.20	14.70	11.70
Values are reported as fold change compared to baseline (V1D0) of the frequency of CD38+ cells within cTFH2 cells.							

Supplementary Table 31 – Number of differentially expressed genes in each pathway following MVA-S vaccination

**Canonical Pathways**	**V1D1**	**V1D3**	**V1D7**	**V2D1**	**V2D7**
Fcgamma receptor (FCGR) dependent phagocytosis	0	0	0	2	0
Immunoregulatory interactions between a Lymphoid and a non-Lymphoid cell	0	0	0	2	0
Fc epsilon receptor (FCERI) signaling	0	0	0	2	0
Signaling by the B Cell Receptor (BCR)	0	0	0	0	0
Interferon alpha/beta signaling	13	1	0	0	0
Interferon gamma signaling	5	0	0	0	0
Phagosome Formation	1	0	0	0	0
Class I MHC mediated antigen processing and presentation	1	0	1	0	0
Neutrophil degranulation	0	0	0	0	0
IL-12 Signaling and Production in Macrophages	0	0	0	0	0
Activation of IRF by Cytosolic Pattern Recognition Receptors	3	0	0	0	0
OAS antiviral response	2	0	0	0	0
ISG15 antiviral mechanism	4	0	0	0	0
Role of PKR in Interferon Induction and Antiviral Response	0	0	0	0	0
Macrophage Classical Activation Signaling Pathway	1	1	1	0	0
CGAS-STING Signaling Pathway	1	0	0	0	0
Role of Pattern Recognition Receptors in Recognition of Bacteria and Viruses	2	0	0	0	0
IL-27 Signaling Pathway	0	0	0	0	0
Complement cascade	1	0	0	2	0
PI3K Signaling in B Lymphocytes	0	0	1	3	0
IL-15 Signaling	0	0	1	3	0
TREM1 Signaling	1	0	0	0	0
Role of NFAT in Regulation of the Immune Response	0	0	1	4	0
Interleukin-10 signaling	2	0	0	0	0
Crosstalk between Dendritic Cells and Natural Killer Cells	0	0	0	0	0
Fcγ Receptor-mediated Phagocytosis in Macrophages and Monocytes	0	0	0	0	0

Supplementary Table 32 – Number of differentially expressed genes in each pathway following MVA-ST vaccination

**Canonical Pathways**	**V1D1**	**V1D3**	**V1D7**	**V2D1**	**V2D7**
Fcgamma receptor (FCGR) dependent phagocytosis	1	0	1	2	4
Immunoregulatory interactions between a Lymphoid and a non-Lymphoid cell	1	0	1	4	3
Fc epsilon receptor (FCERI) signaling	0	0	1	3	3
Signaling by the B Cell Receptor (BCR)	0	0	1	2	2
Interferon alpha/beta signaling	18	0	2	19	0
Interferon gamma signaling	13	0	1	22	0
Phagosome Formation	3	0	0	9	1
Class I MHC mediated antigen processing and presentation	5	0	1	12	1
Neutrophil degranulation	0	0	0	4	0
IL-12 Signaling and Production in Macrophages	4	0	0	7	1
Activation of IRF by Cytosolic Pattern Recognition Receptors	6	0	0	6	0
OAS antiviral response	4	0	1	4	0
ISG15 antiviral mechanism	7	0	0	6	0
Role of PKR in Interferon Induction and Antiviral Response	7	0	0	7	0
Macrophage Classical Activation Signaling Pathway	9	0	0	12	0
CGAS-STING Signaling Pathway	4	0	0	7	0
Role of Pattern Recognition Receptors in Recognition of Bacteria and Viruses	6	0	1	7	0
IL-27 Signaling Pathway	3	0	0	4	0
Complement cascade	1	0	2	5	4
PI3K Signaling in B Lymphocytes	1	0	1	3	7
IL-15 Signaling	0	0	1	1	7
TREM1 Signaling	1	0	0	2	0
Role of NFAT in Regulation of the Immune Response	1	0	1	2	8
Interleukin-10 signaling	2	0	0	3	0
Crosstalk between Dendritic Cells and Natural Killer Cells	0	0	0	1	0
Fcγ Receptor-mediated Phagocytosis in Macrophages and Monocytes	1	0	0	2	0

Supplementary Table 33 – Number of differentially expressed genes in each pathway following ChAd/mRNA vaccination

**Canonical Pathways**	**V1D1**	**V1D3**	**V1D7**	**V2D1**	**V2D3**	**V2D7**
Fcgamma receptor (FCGR) dependent phagocytosis	12	0	64	9	14	19
Immunoregulatory interactions between a Lymphoid and a non-Lymphoid cell	36	0	60	16	13	16
Fc epsilon receptor (FCERI) signaling	14	0	60	9	13	16
Signaling by the B Cell Receptor (BCR)	14	0	53	11	11	15
Interferon alpha/beta signaling	40	2	2	35	7	0
Interferon gamma signaling	41	1	1	34	2	0
Phagosome Formation	87	3	20	38	5	6
Class I MHC mediated antigen processing and presentation	56	0	1	30	1	1
Neutrophil degranulation	74	0	3	38	0	0
IL-12 Signaling and Production in Macrophages	37	1	7	19	2	4
Activation of IRF by Cytosolic Pattern Recognition Receptors	20	1	1	13	1	0
OAS antiviral response	6	1	0	5	1	0
ISG15 antiviral mechanism	14	0	0	11	2	0
Role of PKR in Interferon Induction and Antiviral Response	28	1	1	18	0	0
Macrophage Classical Activation Signaling Pathway	38	1	1	23	0	0
CGAS-STING Signaling Pathway	25	0	1	18	0	0
Role of Pattern Recognition Receptors in Recognition of Bacteria and Viruses	42	5	1	23	4	0
IL-27 Signaling Pathway	26	1	0	22	0	0
Complement cascade	11	5	64	7	19	19
PI3K Signaling in B Lymphocytes	17	1	123	10	24	32
IL-15 Signaling	8	1	123	7	24	32
TREM1 Signaling	22	1	1	17	0	0
Role of NFAT in Regulation of the Immune Response	22	1	123	14	24	32
Interleukin-10 signaling	13	1	1	10	0	0
Crosstalk between Dendritic Cells and Natural Killer Cells	24	0	1	13	0	0
Fcγ Receptor-mediated Phagocytosis in Macrophages and Monocytes	17	0	0	14	0	0

Supplementary Table 34 – Number of differentially expressed genes in each pathway following mRNA vaccination

Canonical Pathways	V1D1	V1D3	V1D7	V2D1	V2D3	V2D7
Fcgamma receptor (FCGR) dependent phagocytosis	0	0	0	4	0	18
Immunoregulatory interactions between a Lymphoid and a non-Lymphoid cell	0	0	0	10	0	16
Fc epsilon receptor (FCERI) signaling	0	0	0	9	0	16
Signaling by the B Cell Receptor (BCR)	0	0	0	9	0	15
Interferon alpha/beta signaling	0	2	1	28	0	6
Interferon gamma signaling	0	0	0	32	0	1
Phagosome Formation	0	0	2	29	2	4
Class I MHC mediated antigen processing and presentation	0	0	0	28	0	0
Neutrophil degranulation	0	0	1	22	0	2
IL-12 Signaling and Production in Macrophages	0	0	0	18	0	2
Activation of IRF by Cytosolic Pattern Recognition Receptors	0	0	0	10	0	1
OAS antiviral response	0	0	0	6	0	1
ISG15 antiviral mechanism	0	0	0	9	0	2
Role of PKR in Interferon Induction and Antiviral Response	0	0	1	14	0	1
Macrophage Classical Activation Signaling Pathway	0	1	1	22	1	1
CGAS-STING Signaling Pathway	0	0	1	15	0	1
Role of Pattern Recognition Receptors in Recognition of Bacteria and Viruses	0	0	0	23	3	1
IL-27 Signaling Pathway	0	0	1	18	0	1
Complement cascade	0	0	0	9	3	19
PI3K Signaling in B Lymphocytes	0	0	1	6	0	26
IL-15 Signaling	0	0	0	4	0	25
TREM1 Signaling	0	0	0	12	0	0
Role of NFAT in Regulation of the Immune Response	0	0	0	8	1	25
Interleukin-10 signaling	0	1	1	7	1	1
Crosstalk between Dendritic Cells and Natural Killer Cells	0	0	1	9	0	0
Fcγ Receptor-mediated Phagocytosis in Macrophages and Monocytes	0	0	1	9	0	0

Supplementary Table 35 – Differentially expressed genes following MVA-S vaccination

V1D1	V1D3	V1D7	V2D1	V2D7
ISG15<br>EXOC3L1<br>CCL2<br>IFIT1<br>SIGLEC1<br>MX1<br>ETV7<br>BATF2<br>CMPK2<br>HERC5<br>RSAD2<br>IFI6<br>IFI35<br>OAS3<br>IFI44L<br>RSPH9<br>CCL8<br>IFIT3<br>OASL<br>RNU7-40P<br>SERPING1<br>EPSTI1<br>IRF7<br>IFIT2<br>LOC102724608<br>CXCL10<br>MT2A<br>XAF1<br>IFIT5<br>PML<br>KDM4D	IFI27	BIRC5<br>CEP55<br>E2F8<br>ESCO2<br>MKI67<br>NCAPG<br>RRM2<br>TOP2A	IGLV2-18<br>IGLV4-60	n.a.

Supplementary Table 36 – Differentially expressed genes following MVA-ST vaccination

**V1D1**	**V1D3**	**V1D7**	**V2D1**	**V2D7**
ETV7	n.a.	DTL	APOL4	DTL
BATF2		CDC20	ANKRD22	RRM2
CCL8		MCM10	BATF2	PBK
CXCL10		RRM2	IDO1	PIMREG
ANKRD22		MKI67	CXCL10	TYMS
APOL4		RSAD2	GBP1P1	IGLV3-27
SERPING1		CDC45	LAMP3	MCM10
LAMP3		SERPING1	ETV7	BIRC5
GBP1P1		KIF20A	CD274	CDC45
CCL2		TYMS	CXCL9	CDC20
LAP3		KIF18B	FCGR1CP	IGLV3-16
SIGLEC1		CCNB2	SERPING1	CDCA5
CD274		OAS3	ATF3	TICRR
GBP1		CDT1	GBP1	E2F8
CMPK2		TICRR	GBP5	CDT1
FCGR1A		BATF2	ETV7-AS1	CDCA2
CALHM6		TOP2A	FCGR1A	SPC25
OAS3		E2F8	P2RY14	FOXM1
ISG15		CDCA2	CALHM6	CCNB2
ATF3		FOXM1	PDCD1LG2	MKI67
FCGR1CP		TK1	ACOD1	TK1
MX1		CDCA5	SOCS1	KIF18B
RSAD2		CKAP2L	LAP3	CDC25A
HERC5		KIF4A	GBP4	IGHG1
CXCL9		BUB1B	IGLV5-45	DLGAP5
OASL		CDC25A	SEPTIN4	BUB1B
IFIT1		BIRC5	WARS1	SKA3
EXOC3L1		CMPK2	GBP6	KIF4A
IFIT3		DIAPH3	SLAMF8	KIF20A
GBP5		TPX2	CCL2	CKAP2L
SLAMF8		DLGAP5	SIMALR	KIFC1
IFI35		SIGLEC1	FCGR1BP	MELK
SEPTIN4		KIFC1	IL31RA	DMC1
PARP14		ETV7	GRIN3A	DEPDC1
UBE2L6		NEK2	AC0123634	TOP2A
GRIN3A		UHRF1	RMI2	TROAP
PDCD1LG2		TROAP	LHFPL2	EXO1
IFI6		NCAPG	LINC02555	ESPL1
IDO1		ESPL1	CMPK2	GINS2
SOCS1		KCNK12	PSTPIP2	GTSE1
STAT2		RP11_319G93	UBE2L6	RAD54L
IFIT2		RNY1	VAMP5	CEP55
SIMALR			PARP14	KIF2C
FCGR1BP			RP11_186N153	FLJ13224
USP18			MYOF	CDC6
PML			FAM225A	UHRF1
WARS1			APOL1	NCAPG
GBP4			IFI35	CDCA7
IFI44L			RP11_44K62	NEK2
EPSTI1			BNIP5	TPX2
SAMD4A			SCARF1	IGHV1-58

IRF7<br>OAS1<br>APOL1<br>LHFPL2<br>SECTM1<br>FAM225A<br>FBXO6<br>IL31RA<br>IL27<br>STAT1<br>IFIH1<br>HELZ2<br>OAS2<br>NRIR<br>MSR1<br>ACOD1<br>XAF1<br>APOL6<br>PARP9<br>IFT56<br>SAMD9L<br>SCARF1<br>EIF2AK2<br>RPL34			OAS3<br>EXOC3L1<br>IL27<br>RSAD2<br>NRN1<br>IDO2<br>EPSTI1<br>FBXO6<br>FRMD3<br>CCR5AS<br>APOL6<br>FZD5<br>SECTM1<br>ISG15<br>SHOX2<br>EFCAB2<br>CXCL11<br>IFIT3<br>STAT1<br>CEACAM1<br>STAT2<br>PML<br>SAMD9L<br>AC008496.3<br>CCL8<br>SAMD4A<br>KCTD14<br>GBP7<br>ENSG00000289582<br>FAM20A<br>RN7SKP26<br>CIBAR1<br>XXYLT1-AS2<br>PLSCR1<br>TGM2<br>KLF5<br>PLSCR4<br>IRF7<br>TAP1<br>OASL<br>TNFAIP6<br>CCRL2<br>PARP9<br>LPCAT2<br>HERC5<br>TIFA<br>IFI44L<br>VPS9D1<br>SIGLEC1<br>IFT56<br>IFIH1<br>IRF1<br>IFI6<br>GK-IT1	IGKV1-16<br>HJURP<br>CCNA2<br>CLSPN<br>HPDL<br>CDCA3<br>TEDC2<br>ASPM<br>RAD51<br>PKMYT1<br>CENPM<br>NEIL3<br>CDK1<br>SPC24<br>IGLV3-9<br>ZWINT<br>MYBL2<br>SKA1<br>TMEM30A-DT<br>RPL29P24<br>RP11_338E212<br>EEF1B2P6<br>RNY1

			C3<br>GBP2<br>PSME2P2<br>GSDMC<br>GPR84<br>XAF1<br>CIMAP1B<br>PTGES3P1<br>SCO2<br>SLC6A12<br>TRIM22<br>PRRG4<br>GCH1<br>RNU7-40P<br>RHBDF2<br>DTX3L<br>SORT1<br>HELZ2<br>LINC02471<br>RTP4<br>OAS1<br>CAPNS2<br>APOL2<br>LYPD5<br>CFAP97D1<br>ENSG00000288836<br>MX1<br>SMCO4<br>LOC102724608<br>TRAFD1<br>CCDC194<br>HCAR1<br>ICAM1<br>AC092428.1<br>SDC3<br>NUCB1<br>GK<br>PSME2<br>LINC02068<br>AL353807.5<br>XRN1<br>MIR194-2HG<br>RP11_242C192<br>STX11<br>GRAMD1B<br>CTD_2639E64<br>TMEM140<br>CARD17P<br>GK4P<br>RNF213<br>ASPHD2<br>AIM2<br>IFIT2<br>RAB20	

			MICB-DT<br>KIF24<br>GBP3<br>TYMP<br>FAS<br>BCL2L14<br>OAS2<br>RP11_329N153<br>NCF1B<br>RUFY4<br>RPS29P14<br>KREMEN1<br>HMGA2-AS1<br>C2<br>DDX60<br>SPATS2L<br>TAP2<br>MED12L<br>NEXN<br>TMEM150B<br>SUCNR1<br>HTR3B<br>RAB39A<br>ERLIN1<br>IFI44<br>PSMB9<br>MAFF<br>DOCK4<br>LINC01232<br>MOV10<br>LACTB<br>IFIT1<br>TRIM21<br>PRKCG<br>STK3<br>BISPR<br>CDCP1<br>MT2A<br>FANCA<br>AP001977.1<br>RP3_330M215<br>RNY1	
